# Photochemical Aging of Indole SOA: Implications for Volatility and Optical Properties

**DOI:** 10.1021/acs.est.5c10237

**Published:** 2026-02-24

**Authors:** Thenoor Chandran Ajith, Diego Calderon-Arrieta, Hongwei Pang, Zheng Fang, JingKai Wang, Jessica Knull, Nyiri Hajian, Kirby Hill, Chunlin Li, Alexander Laskin, Yinon Rudich

**Affiliations:** † Department of Earth and Planetary Sciences, 34976 Weizmann Institute of Science, Rehovot 7610001, Israel; ‡ Department of Chemistry, 311308 Purdue University, West Lafayette, Indiana 47907, United States; § College of Environmental Science and Engineering, Tongji University, Shanghai 200092, China; ∥ Department of Earth, Atmospheric and Planetary Sciences, Purdue University, West Lafayette, Indiana 47907, United States

**Keywords:** brown carbon, optical properties, thermodenuder, volatility

## Abstract

This study investigates the chemical composition, volatility, and optical properties of secondary organic aerosol (SOA) formed from 1 and 5 days of equivalent photochemical oxidation of indole using a combination of online thermodenuder techniques and offline high-resolution mass spectrometry (HRMS). Thermodenuder–Aerosol Mass Spectrometer (TD-AMS) thermograms revealed higher volatility for CH and CHO fragments (*T*_50_ ∼ 390–395 K) and greater thermal stability for nitrogen-containing CHON ions (*T*_50_ ∼ 410–415 K). Volatility basis set (VBS) distributions showed that CHON species dominated the composition of 1-day aged SOA (INDOH1) but were largely depleted in 5-day aged SOA (INDOH5), indicating extensive oxidative aging associated with ring-opening reactions and the loss of nitrogen-containing functional groups, as reflected in the degradation of aromatic species (AI_mod_ > 0.67) and reduced π-conjugation. Additionally, INDOH1 exhibited stronger light absorption than INDOH5, demonstrating significant photobleaching. The evaporation due to heating affected the complex refractive index (RI); the imaginary part (*k*) increased from 0.014 to 0.09 in INDOH1, while it remained below 0.06 in INDOH5. The absorption enhancement with heating is attributed to the preferential evaporation of weakly absorbing, nonaromatic compounds, enriching the particle phase in thermally stable, π-conjugated CHON species. These results establish a direct link between the volatility, chemical evolution, and optical properties of indole SOA.

## Graphical abstract

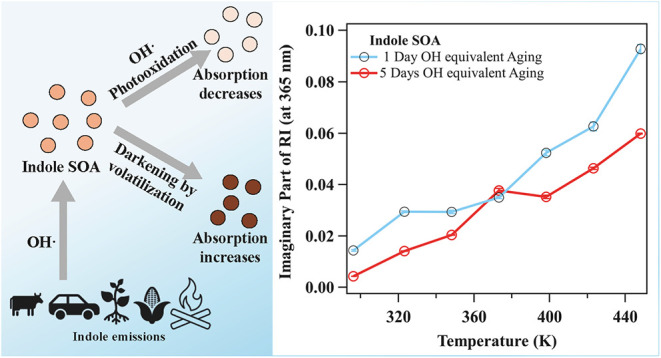

## 1 Introduction

Atmospheric aerosols have a significant impact on Earth’s climate^1^ and pose risks to human health.^2^ Organic aerosols (OA) constitute a substantial fraction of submicron tropospheric particles and present considerable challenges in accurately quantifying their climate impacts.^3,4^ OA can be either directly emitted as primary organic aerosol (POA) or formed through the atmospheric oxidation of volatile organic compounds (VOCs), producing secondary organic aerosol (SOA).^5^ Recently, the formation and evolution of SOA in the atmosphere have received greater attention due to its substantial contribution to total OA mass and its influence on climate-relevant aerosol properties.^6^

The volatility of OA, commonly expressed as the effective saturation mass concentration, C* [μg m^–3^], is an important physicochemical property that determines the partitioning of the organic compounds between the gas and particulate phase. This, in turn, strongly influences OA mass concentration, chemical composition, and the size distributions.^3,7–9^ Comprehensive characterization of OA volatility, both in ambient and laboratory settings, is essential for improving the mechanistic understanding and for accurate modeling of OA formation and atmospheric evolution.^5,7^ The one-dimensional volatility basis set (VBS) framework, developed by Donahue et al.,^10^ categorizes OA components across a range of volatility (C*) values (from <10^–4^ to 10^6^ μg m^–3^) using logarithmically spaced bins. This framework has since been extended into multidimensional VBS schemes that incorporate additional chemical characteristics such as oxidation state and molecular functionality.^3,7,11^ The VBS framework is widely employed in both experimental measurements^12,13^ and atmospheric modeling studies.^14–17^ Thermal desorption methods are commonly used to quantify OA volatility in laboratory and field studies. A thermodenuder (TD), often coupled with scanning mobility particle sizer (SMPS) and aerosol mass spectrometer (AMS), enables temperature-resolved analysis of OA evaporation.^9,18,19^ In this setup, particles are exposed to a controlled temperature ramp inside the TD, and the mass fraction remaining (MFR) is measured as a function of the temperature. Several parametrizations and kinetic models have been developed to interpret TD-based data and to derive OA volatility distributions.^20–22^

There is a growing interest in understanding the light-absorbing components of OA known as brown carbon (BrC).^23–25^ In the atmosphere, BrC undergoes oxidative aging, which modifies its optical properties over time.^23,26–30^ During transport from their emission sources, BrC also experience dilution, leading to the loss of higher-volatility compounds. This loss can affect particle absorption depending on the optical characteristics of both the evaporated species and the residual material in the condensed phase.^31,32^ Thus, both oxidative aging and dilution-driven evaporation can significantly influence the absorbing nature of BrC, although the extent and direction of these effects are uncertain.^24^ While studies examining the influence of oxidative aging on the optical properties of BrC are available, the role of volatility and its connection to optical properties remain sparse, highlighting the need for further investigations.

Indole is a nitrogen-containing heterocyclic VOC from various atmospheric sources. It is emitted by both biogenic and anthropogenic sources, including biomass-burning, engine exhausts,^33–35^ animal husbandry,^36^ and agricultural plants such as rice and maize.^37,38^ Plants emit indole, particularly during periods of physiological stress and flowering. Global emissions and emission factors of indole are estimated at approximately 0.1 Tg yr^–1^ and 0.6 μg m^–2^ h^–1^, respectively.^38^ Field measurements report mixing ratios ranging from 1–2.7 ppb during daytime to 1.5–3.7 ppb at nighttime, often exceeding isoprene levels during spring flowering events.^39^ In the San Joaquin Valley, California, indole concentrations reached 4.7–18 μg m^–3^, comparable to or higher than those of the dominant monoterpene myrcene.^39^ Indole and its derivatives have long been used in agriculture and consumer products, including a well-known derivative, indigo dye.^40^

Considering the importance of indole, several recent studies have explored the physical and chemical properties of indole SOA formed from the major atmospheric oxidants OH^•^, O3^•^, NO3^•^, and Cl^•^, highlighting the higher SOA yields and their contribution to BrC components.^30,41–43^ The reported SOA mass yield was 1.3 ± 0.3, and the mass absorption coefficient (MAC_300_ nm) reached approximately 2 m^2^ g^–1^, indicating strong light absorption in the near-UV region. The absorption Ångström exponents (AAE) were 6.8 ± 0.2 and 5.83 ± 0.3 for the 375–550 and 315–450 nm wavelength ranges, respectively.^42^ Despite these advancements, to our knowledge, no studies so far investigated the volatility of indole SOA and its relation to photochemical aging and optical properties.

In this work, a TD is employed to evaporate volatile components from indole SOA generated in a potential aerosol mass (PAM) oxidation flow reactor (OFR) under various OH aging conditions, and the resulting changes in the physical and chemical properties are investigated. Here, TD provides a laboratory analog to volatility-driven evaporation that occurs under atmospheric dilution and transport. This experimental approach enables controlled probing of the link between volatility and chemical and optical properties, offering insights relevant to the atmospheric evolution of brown carbon. Additionally, filter samples of indole SOA were collected at room temperature for advanced chemical analyses, and VBS distributions for indole SOA were constructed. The evolution of the complex refractive index (RI) of indole SOA with heating is also retrieved, providing new insights into the relationship between volatility and optical properties.

## 2 Experiments and Instruments

### 2.1 Indole SOA Generation

Figure 1 shows the experimental setup for the measurements. The indole SOA was generated from OH^•^ photooxidation in a PAM-OFR (Aerodyne Research, In., MA). A detailed working principle of the PAM-OFR can be found elsewhere.^27,44,45^ UV irradiation at 254 nm was produced inside the chamber with two controllable mercury lamps. Ozone and H_2_O are supplied externally; the UV light photolyzes the ozone to form O^1^D and then reacts with H_2_O to form OH^•^. An ozone monitor (Model 106-L, 2B Tech) continuously detected the ozone concentrations during the experiment. A total flow rate of 4.5 L min^–1^ with a residence time of about 170 s was maintained during the experiments. Gaseous indole (∼545 ppb) was generated by passing a gentle stream of nitrogen (∼35 sccm) over solid indole (50 mg of indole powder, purity ≥99.0%, Sigma-Aldrich) contained in an impinger immersed in a hot-water bath (temperature ∼43 °C). The extent of indole SOA aging, determined by the OH^•^ exposure (OH exp), was controlled by modifying the UV light intensity and the ozone depletion ratio following the recommended manufacturer instructions (https://sites.google.com/site/pamwiki/estimation-equations). The aging time scales were calculated by assuming a daily average OH^•^ concentration of 1.5 × 10^6^ molecules cm^–3^.^46^

**Figure 1:**
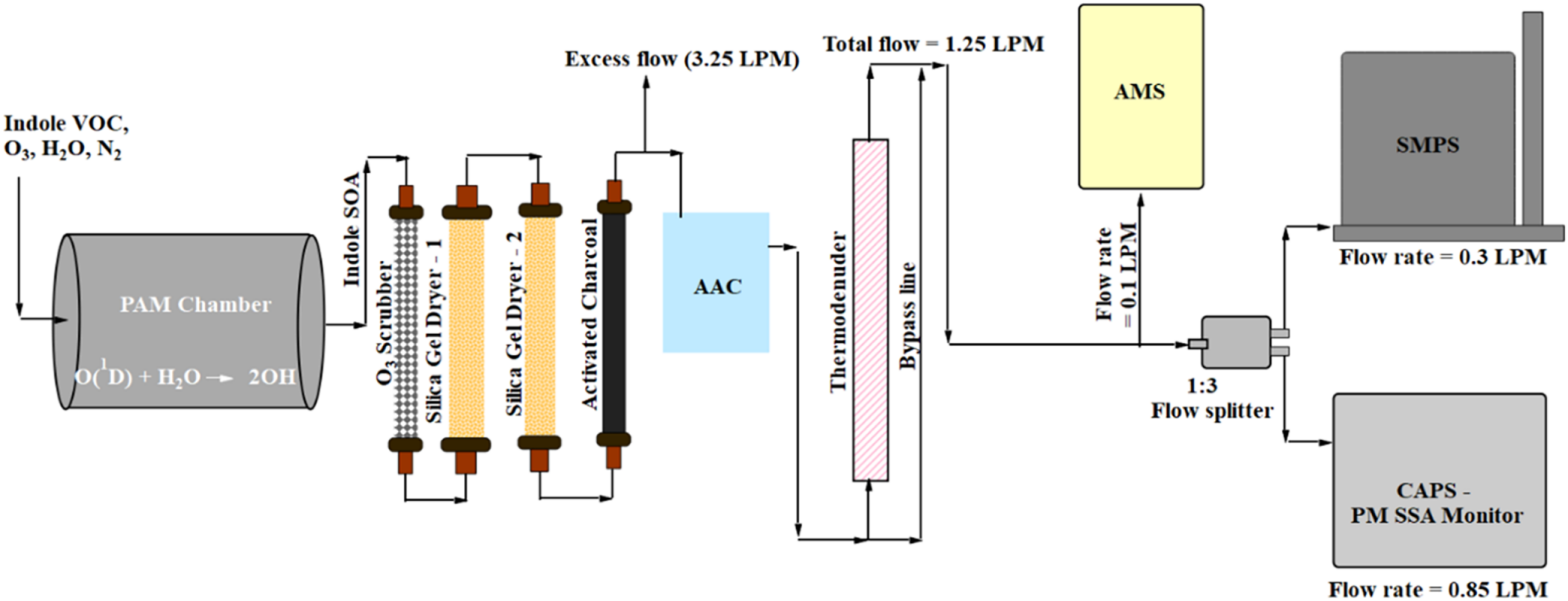
Schematic of the experimental setup for generating and measuring indole SOA. The Potential Aerosol Mass (PAM) chamber generates indole SOA, and an Aerodynamic Aerosol Classifier (AAC) size-selects the particles to obtain an SMPS-measured mobility diameter of 100 nm. The size-selected particles pass alternately through a thermodenuder or a bypass line. The chemical composition, particle size distribution, and optical coefficients (scattering and extinction) are measured using an Aerosol Mass Spectrometer (AMS), a Scanning Mobility Particle Sizer (SMPS), and a Cavity Attenuated Phase-Shift Single-Scattering Albedo (CAPS SSA) monitor.

The generated indole SOA downstream of the PAM-OFR passed through a set of denuders containing ozone scrubbers, activated charcoal, and silica gel before physical and chemical characterization. Of the total 4.5 L min^–1^ exiting the PAM-OFR, 1.25 L min^–1^ was drawn by the sampling instruments as shown in Figure 1, while the remaining 3.25 L min^–1^ was vented to the exhaust line. The dried particles were size selected using an aerodynamic aerosol classifier (AAC, Cambustion) to obtain an SMPS-measured mobility diameter of 100 nm. The number size distributions, optical properties, and volatility of the size-selected particles were measured subsequently using a TD coupled with an SMPS, CAPS, and AMS.

### 2.2 Thermodenuder

We used a commercial TD (Brechtel Ltd. TD 3105) that has previously been described.^47,48^ The TD comprises three sections: (1) a heating zone, (2) a residence zone, and (3) a denuder and cooling zone. In the heating zone, two opposing heating elements heat the sample to the temperature set point, and the sample moves toward the residence zone; the set temperature is uniformly maintained throughout the residence zone. The temperature is controlled using a thermistor, with a maximum setting of 573.15 K. The residence time is 38 and 20 s for 298.15 and 573.15 K (±0.1 K), respectively, at a flow rate of 1 LPM. In this study, a programmed temperature ramp was used with set points of 296.15 K (room temperature), 323.15, 348.15, 373.15, 398.15, 423.15, and 448.15 K. At each temperature, the system was held for ∼40 min, and only data from the final 15 min, during which the temperature was stable, were used for analysis. The wall loss and thermophoresis loss for the TD were estimated using nebulized NaCl particles following the methodology described in the study by Huffman et al.^49^ NaCl particles were generated using a collision-type atomizer (Model: 3076, TSI), dried using a silica gel-based dryer, diluted, and size selected using AAC to obtain an SMPS-measured mobility diameter of 100 nm. The size-selected particles were switched between the TD and the bypass line, and the number size distributions were measured by using a scanning mobility particle sizer (SMPS, TSI Incorporated, Classifier Model: 3080, DMA 3081, and CPC 3775). The cavity attenuated phase-shift single-scattering albedo monitor (CAPS PM SSA monitor, Aerodyne Research, Inc., MA, later known as “SSA monitor”) at 365 nm^50^ was also connected in parallel with SMPS to maintain the flow rate used in the chamber experiments. Under room temperature (∼296.15 K), negligible loss (∼1%) for particle number and mass was observed between the bypass line and TD. The particle mass losses observed for 100 nm particles were 8% (323.15 K), 20% (348.15 K), 23% (373.15 K), 24 ± 3% (398.15 K), 25 ± 3% (423.15 K), 29% (448.15 K). All TD thermograms in this study were corrected following the values described above. Similar values for mass loss corrections were previously reported by Huffman et al.^49^ and Chen et al.^51^

### 2.3 Particle Number Size Distributions, Chemical Composition, and Optical Properties

Aerosol size distributions and chemical compositions of indole SOA were measured using an SMPS and High-Resolution Time-of-Flight Aerosol Mass Spectrometer (HR-ToF-AMS, Aerodyne Research, Inc., MA). The HR-ToF-AMS was operated in both V and W modes alternately, with data analysis conducted using the analysis software packages Squirrel (version 1.65) and PIKA (version 1.25) in IGOR Pro (version 6.3.7.2). A detailed description of the HR-ToF-AMS operating principles can be found in previous studies.^52,53^ Organic elemental ratios of O/C, H/C, and N/C were derived from the relative intensities of organic ion fragments in the HR-AMS mass spectra following the methodology described by Canagaratna et al.^52^

A newly designed SSA monitor at 365 nm (described in detail by Ajith et al.^50^) was used to measure the optical properties of the indole SOA. The SSA monitor provides real-time scattering and extinction coefficients at a wavelength of 365 nm. The complex refractive index (RI) of indole SOA was determined from the scattering coefficients and absorption coefficients (derived via the EMS method^54^) along with SMPS measurements. A detailed explanation of the working principle of the SSA monitor and the methodology followed in retrieving the complex refractive index is given in Supporting Note A.

### 2.4 Offline Chemical Analysis, Optical Measurements of Filter Samples, and Molecular Characterization

Quartz-fiber filters positioned downstream of the room-temperature-programmed TD were used to collect the indole SOA samples for offline optical and chemical measurements. The preconcentrated filter extracts were analyzed using a hyphenated platform consisting of UltraHigh-Performance Liquid Chromatography (UPLC), photodiode array detection (PDA), and high-resolution mass spectrometry (HRMS). Further details on the offline chemical analysis and instrument parameters are provided in Supporting Notes B and C. The UV light absorbance per unit of organic mass is defined as the mass absorption coefficient, MAC (λ),^55^ which was calculated from the LC-PDA records. Detailed methodology is provided in Supporting Note D. MZMine 2.53 was used to extract ion chromatograms from the background-subtracted high-resolution mass spectra, and elemental formulas were assigned by using established workflows. Accurately assigned species were categorized into the CHO, CHN, and CHON classes. Double bond equivalency (DBE), saturation mass concentrations (*C*_298 K_*), and modified aromaticity index (AI_mod_) were calculated from the elemental formulas of the corresponding neutral molecules. DBE reflects the degree of molecular unsaturation, while AI_mod_ builds on the DBE and provides a measure of conjugated π-bond networks in the detected species. Volatility values and enthalpies of transition were subsequently used to construct representative VBS distributions for the aged indole SOA systems, enabling visualization of their gas-phase and particle-phase composition. Detailed explanations on the molecular characterization and saturation mass concentration and enthalpy of vaporization calculations for indole SOA VBS distributions are given in Supporting Notes E and F.

## 3 Results and Discussion

### 3.1 Physical and Experimental Properties of Indole SOA at Room Temperature

Table 1 shows the conditions maintained in the PAM-OFR and the properties of the indole SOA. The relative humidity (RH ∼ 34–35%), initial ozone concentration (51–52 ppm), and concentration of indole VOC (∼545 ppbv) were constant for all of the experiments. Indole SOA from two aging conditions was generated, the first with 1 day equivalent atmospheric aging (INDOH1, corresponding to an OH^•^ exposure of 1.3 × 10^11^ cm^–3^ and O_3_ exposure of 1.6 × 10^17^ cm^–3^) and the second with 5 days equivalent atmospheric aging (INDOH5, corresponding to an OH^•^ exposure of 6.5 × 10^11^ cm^–3^ and O_3_ exposure of 8.8 × 10^16^ cm^–3^). Considering the higher reactivity of OH^•^ (reaction constant ∼ 1.5× 10^–10^ cm^3^ molecules^–1^ s^–1^) toward indole compared to O_3_ (reaction constant ∼ 1.3× 10^–17^ cm^3^ molecules^–1^ s^–1^), the oxidation processes in this study are expected to be dominated by OH^•^-initiated reactions, with only minor contributions from O_3_, which were not considered further. Figure S4 shows the AMS mass spectra for INDOH1, and Figure S5 shows the same for INDOH5. The AMS mass spectra of indole SOA resembles those reported by Li et al.^42^ showing a contribution from C_*x*_H_*y*_^+^, C_*x*_H_*y*_O^+^, C*_x_* H*_y_* O*_z_* ^+^, C*_x_* H*_y_* N^+^, and C*_x_* H*_y_* ON^+^ families. With increasing OH^•^ exposure from 1 to 5 days, the relative intensity of C*_x_* H*_y_* O^+^ decreases, while more oxygenated C*_x_* H*_y_* O*_z_* ^+^ increases, indicating prolonged oxidation. The mass spectrum reported by Li et al. (∼3.5 days of OH equivalent aging) exhibit intermediate characteristics, falling between the 1-day and 5-day spectra in this study. A clear enhancement in the O:C ratio (increased from 1.14 to 1.53) derived from AMS measurements with higher aging is reflected. Further, the H:C ratio decreased from 1.06 ± 0.02 to 0.86 ± 0.02. The effective density of indole SOAs was determined using the aerodynamic diameter measured by the AAC and the mobility diameter obtained after the AAC with an SMPS, as described in the study by DeCarlo et al.^56^ The effective density of indole SOA increased upon aging from 1.47 ± 0.01 to 1.52 ± 0.01 g cm^–3^, consistent with the general trend of increasing density with oxidative aging. The densities derived in this study are comparable to those of the indole oxidation products: isatin (1.47 g cm^–3^), anthranilic acid (1.40 g cm^–3^), and isatoic anhydride (1.52 g cm^–3^).

**Table 1:** Conditions inside PAM-OFR and Properties of Generated Indole SOA

	PAM-OFR conditions			indole SOA properties		
experiment	concentration of VOC (ppbv)	relative humidity	ozone (ppm)	effective density (g cm^–3^)	O:C	H:C
indole SOA—1-day equivalent OH aging (INDOH1)	545	35.0%	51.4 ± 0.5	1.47 ± 0.01	1.14 ± 0.02	1.06 ± 0.02
indole SOA—5-days equivalent OH aging (INDOH5)	545	34.6%	52 ± 0.5	1.52 ± 0.01	1.53 ± 0.03	0.86 ± 0.02

### 3.2 Volatility Characterization Using TD Mass Thermograms

The mass fraction remaining (MFR) was calculated as the ratio of the mass of indole SOA particles exiting the TD to the mass in the bypass line. Figure 2 shows the MFR versus TD temperature scatter plot (mass thermograms) measured during this study. The data points were fitted using the sigmoidal eq 1 described in Kolesar et al.^57^ and Emanuelsson et al.^58^ and a similar temperature-dependent MFR fitting approach has also been applied in Li et al.^59^

1 $$MFR\;\left(T\right)=\left(\frac{{MFR}_{\min}-{MFR}_{\max}}{1+{\left(\frac{T}{T_{50}}\right)}^{-S}}\right)$$

where *S* is the slope of the MFR curve and *T*_50_ is the TD temperature at which MFR = 0.50. The upper and lower asymptotes, MFR_max_ and MFR_min_, are assumed to be 1 and 0, respectively.

**Figure 2:**
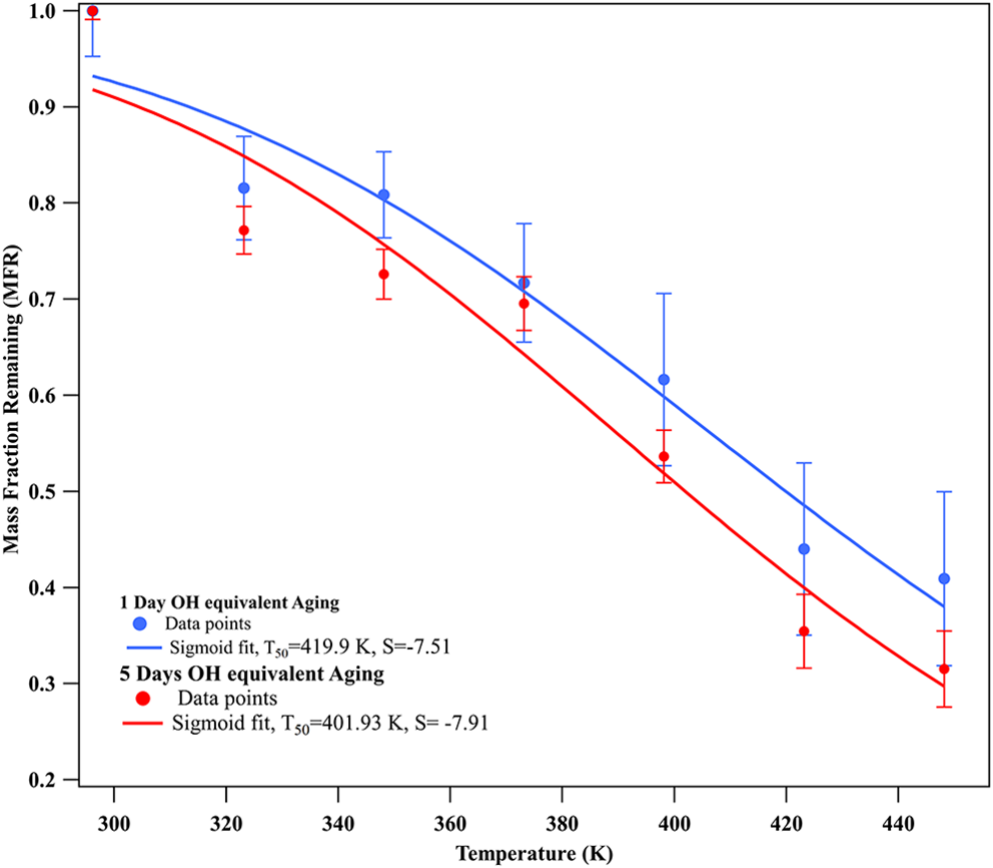
Mass thermograms of indole SOA subjected to 1 day (INDOH1) and 5 days (INDOH5) of equivalent OH equivalent aging. Sigmoidal fits were applied using eq 1 to determine volatility parameters.

The mass thermograms for INDOH1 and INDOH5 followed similar patterns, but the differences in *T*_50_ values indicate a clear effect of oxidative aging on the volatility. A rapid reduction (∼20%) in MFR for INDOH1 and INDOH5 was observed when the temperature increased from 296.15 K (room temperature) to 323.15 K, indicating the evaporation of semivolatile species due to the vapor stripping inside the TD. Additional temperature steps between 296 and 323 K could not be explored due to TD stability constraints, limiting resolution in the semivolatile range. The less-aged SOA (INDOH1) retained a higher MFR at elevated temperatures, indicating lower overall volatility compared to INDOH5. This is evident in the *T*_50_ values and the steepness (*S*) of the sigmoid fit, where INDOH1 had a higher *T*_50_ of 419.9 K with *S* = −7.51, compared to INDOH5, which had a *T*_50_ of 401.93 K and *S* = −7.91. Although the MFR is a relative quantity, its temperature dependence under identical experimental conditions provides a valid basis for comparing volatility trends between samples. The explanation for the higher volatility observed in INDOH5, along with supporting compositional changes, is discussed in Sections 3.3 and 3.4.

### 3.3 Volatility Distributions from Molecular Composition and VBS Framework

Figure 3 illustrates the estimated log_10_(*C*_298 K_*) of the individual components identified in INDOH1 and INDOH5 mixtures from ESI­(+) ionization. Both INDOH1 and INDOH5 samples feature a broader range of chemical constituents, spanning from the intermediately volatile organic compounds (IVOC, 6.48 > log_10_(C*, μg/m^3^) > 2.48) to the extremely low-volatility organic compounds (ELVOC, log_10_(C*, μg/m^3^) < −3.53).^11,60^ However, INDOH5 is dominated by species in the semivolatile organic compound range (SVOC, 2.48 > log_10_(C*, μg/m^3^) > −0.53) and the low-volatility organic compound range (LVOC, −0.53 > log_10_(C*, μg/m^3^) > −3.53). This is consistent with atmospheric OH aging mechanisms, because the relatively fresh INDOH1 precursors are converted into more oxygenated INDOH5-detected species,^61^ thereby decreasing their log_10_(C*, μg/m^3^) values.^62^ The significant absence of CHON species in INDOH5 underscores the susceptibility of these compounds class to photochemical aging mechanisms.

**Figure 3:**
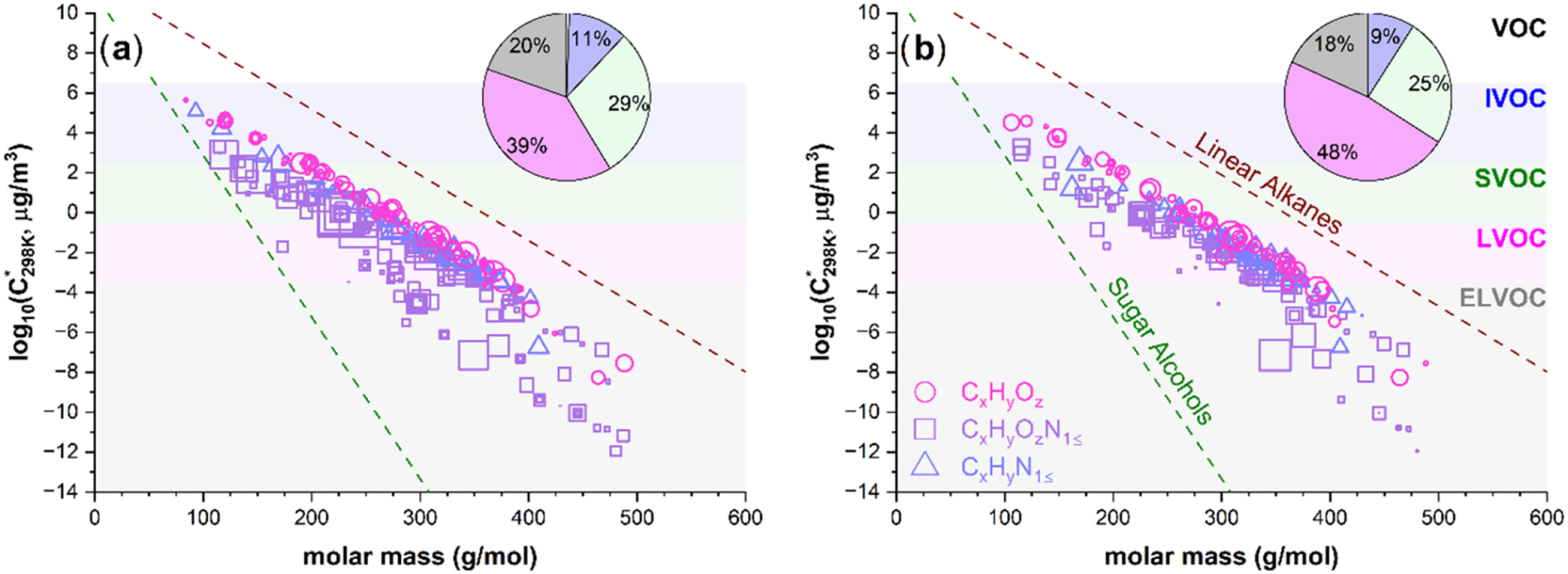
Estimated saturation mass concentration plots for (a) INDOH1 and (b) INDOH5 samples. Background colors denote the five volatility categories: VOC, IVOC, SVOC, LVOC, and ELVOC. Assigned compounds are color-coded based on compound class: CHO (pink circles), CHON (violet squares), and CHN (blue triangles). Pie charts illustrate the relative abundance of species associated with each of the five volatility categories. The cluster of IVOC/SVOC compounds in INDOH1 shifts toward the LVOC category in INDOH5, highlighting the presence of more chemically inert indole SOA species after an extended OH exposure period.

Figure 4 presents VBS distributions constructed for INDOH1 and INDOH5 mixtures from the estimated volatilities and mass fractions of their individual components. The broad distribution of log_10_(*C*_298 K_*, μg/m^3^) values observed in INDOH1 accounts for the wide range of organic mass concentrations comprising VBS. In contrast, the narrower and more centered VBS distribution of INDOH5 sample is consistent with the corresponding compact saturation mass concentration profile shown in Figure 3b. A clear cluster of multiple LVOC species and some sparse yet intense ELVOC species in Figure 3b are reconstructed as tall LVOC peaks in Figure 4b, with some smaller, isolated ELVOC peaks as well. The extent of gas-particle partitioning shown in the plots for both samples was calculated based on total organic mass (tOM) loadings of 20 and 10 μg/m^3^, corresponding to the concentrations measured in the PAM-OFR experiments for INDOH1 and INDOH5, respectively. For both samples, the data indicate a dominant contribution from particle-phase species at room temperature with only trace levels of gas-phase components.

**Figure 4:**
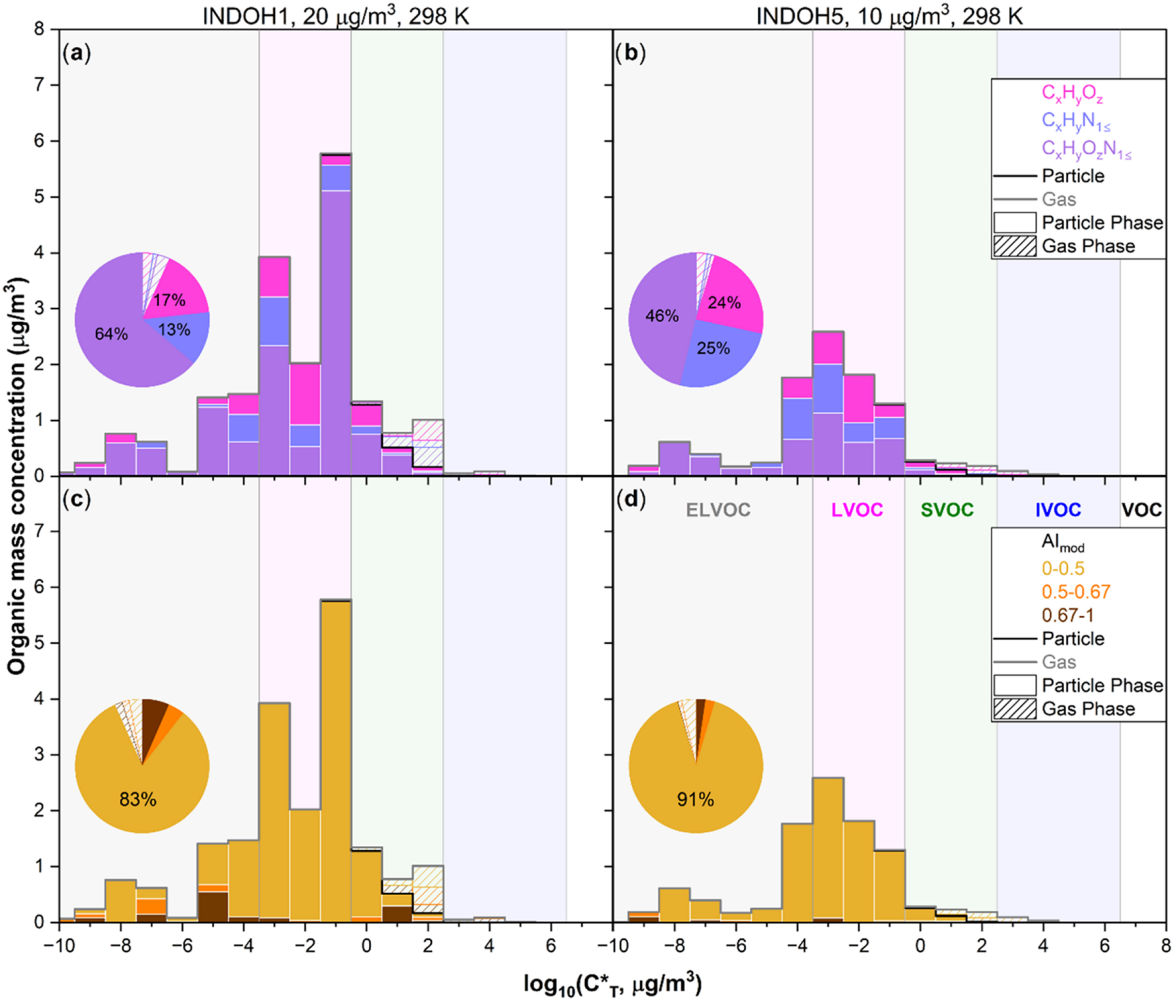
VBS distributions resolved by compound class and AI_mod_ for (a, c) INDOH1 and (b, d) INDOH5. The five VOC categories are denoted by the background colors. (a, b) Panels and corresponding pie graphs convey the total gas-phase and particle-phase abundances of the three compound classes. (c, d) Panels and corresponding pie graphs display the total gas-phase and particle-phase abundances of the three AI_mod_ bins. The VBS distributions together indicate that prolonged 5-day equivalent OH aging consumes CHON compounds and generates fewer aromatic species.

These distributions are narrow compared to other VBS distributions developed for limonene-, α-pinene-, and ocimene-SOA generated via ozonolysis reported in a previous study.^63^ INDOH1 and INDOH5 VBS distributions are also very narrow compared to fresh wood tar condensate (PO_1_ in Xie et al.^64^) and aged wood tar (PO_3_ in Xie et al.^64^). Both samples are characterized predominantly by only two categories: LVOC and ELVOC species. INDOH1 contains 58% LVOC and 24% ELVOC, whereas INDOH5 comprises 57% LVOC and 34% ELVOC. This is due to three major factors: (1) the use of ozone oxidation, a different aging mechanism used to generate the terpene-based SOA samples, (2) the significant fraction of CHO constituent molecules present in the wood tar and terpene-based SOA, and (3) the application of DART(−) ionization for both samples.^63,64^ First, OH^•^- and ozone-generated aerosol components are anticipated to differ profoundly due to their distinct oxidation mechanisms. Ozone-facilitated reactive pathways promote oligomerization via Criegee intermediates,^5^ resulting in pronounced shifts toward less-volatile VBS bins. Conversely, OH^•^ can react with multiple functional groups, yielding both fragmented and oligomerized products.^61^ Second, the abundant presence of CHO compounds associated with wood tar- and terpene-based SOA indicates highly oxidized molecules with large O/C ratios,^5^ further enhancing particle-phase VBS peak heights. In contrast, indole precursor molecules may gain relatively fewer O and C atoms (3-oxindole, isatin, anthranilic acid, and so on)^43^ from oxidation, causing VBS bins in the INDOH1 and INDOH5 systems to cluster across 2–3 bin groups. ESI­(+) ionizes polar CHO species and CHON compounds,^65^ unlike direct analysis in real-time [DART(−)] ionization, which selectively ionizes carboxylic acid-containing species.^66,67^ One additional N in the molecular formula of a compound decreases its expected C_298 K_^0^ (μg/m^3^) by at least an order of magnitude^68^ or an entire bin class shift in a VBS distribution. These VBS plots demonstrate the profound impact that N-heteroatoms have on the formation and condensation of N-containing organic aerosols.

Figure 4a,b presents the VBS distributions resolved by major compound classes: CHN, CHON, and CHO. In INDOH1, CHON species dominate, consistent with the LVOC species that retain nitrogen functionality. In contrast, INDOH5 exhibits a pronounced decay in CHON abundance (64% in the INDOH1 pie graph to 46% in the INDOH5 pie graph) coupled with a buildup of CHN and CHO species. This shift reflects extensive oxidation and molecular fragmentation under prolonged OH exposure, which results in a loss of nitrogen moieties and enhanced oxygenation. Similar trends in the volatility evolution have been reported by Li et al.,^69^ who showed that SOA compounds with higher molecular weight and more functional groups tend to populate lower-volatility (LVOC–ELVOC) bins.

Figure 4c,d presents the VBS data sets resolved by compounds that exhibit AI_mod_ values within three ranges: 0–0.5, 0.5–0.67, and 0.67–1, which highlights the role of π-conjugated structures in governing light-absorbing behavior. Less aromatic constituents (AI_mod_ < 0.5) dominate the SOA, while more aromatic constituents (AI_mod_ > 0.67) are present at trace levels in the INDOH1 mixture. Figure S6 showcases this pattern more clearly. ESI­(+) high-resolution mass spectra of INDOH1 (a) and INDOH5 (b) samples are color-coded based on the AI_mod_ value. Only a few aromatic compounds are present in INDOH1, whereas the INDOH5 system is primarily composed of less aromatic species. Hydrogen at the C(3) position in the pyrrole ring of indole is susceptible to hydroxyl radical H-abstraction, subsequently leading to pyrrole ring-opened products that are less aromatic than their precursors.^70,71^ Most aromatic species that withstood the 1-day equivalent OH exposure further broke down after longer OH exposure, as observed by the slight buildup of nonaromatic species in the INDOH5 mixture. The significant abundance of nonaromatic constituents in both samples is anticipated, because the OH oxidation mechanism reduces aromaticity in aerosol components and whitening the aerosol particles.^23^ While these VBS diagrams lack contributions from nonpolar or acidic CHO compounds due to limited ionization efficiency in ESI­(+), this mode still provides a representative view of the principal polar CHO, CHN, and CHON SOA components formed from indole oxidation.

Figure S7a,b shows the plots of assigned peaks of INDOH1 and INDOH5 samples detected in their respective ESI­(+) high-resolution mass spectra. As Figure S7a shows, four plausible structures, 3-oxindole, anthranilic acid, isatin, and isatoic anhydride, were detected in the INDOH1 system. These species are not detected after prolonged OH aging in the INDOH5 system, as Figure S7b shows. These oxidation products are consistent with those reported by Montoya-Aguilera et al.,^43^ who identified isatin (C_8_H_5_O_2_N) and isatoic anhydride (C_8_H_5_O_3_N) as dominant monomeric species and 3-oxindole (C_8_H_5_ON) as a secondary product.

Based on the observed products in the present study, an OH^•^ initiated photooxidation mechanism for indole is proposed (Supporting Note G). OH^•^ oxidation primarily involves attack on the pyrrole ring via hydrogen abstraction and/or OH addition, leading to carbonyl formation and ring-opening products. These pathways account for the observed decrease in aromaticity (AI_mod_) and the disappearance of these products in the more oxidized INDOH5 system. The proposed reaction mechanism is consistent with that reported by Jiang et al.^70^ for OH^•^ initiated indole oxidation under low-NO*_x_* conditions.

### 3.4 Chemical Family–Resolved Volatility Profiles from TD-AMS and VBS Analysis

Figure 5 shows the TD-AMS-measured (panels a and b) and VBS-derived (panels c and d) thermograms for different chemical families in INDOH1 and INDOH5. These figures reflect the volatility behavior of the chemical families within each sample. Further, gas-particle partitioning trends unveiled by the VBS distributions at different temperatures are explained in Supporting Note H.

**Figure 5:**
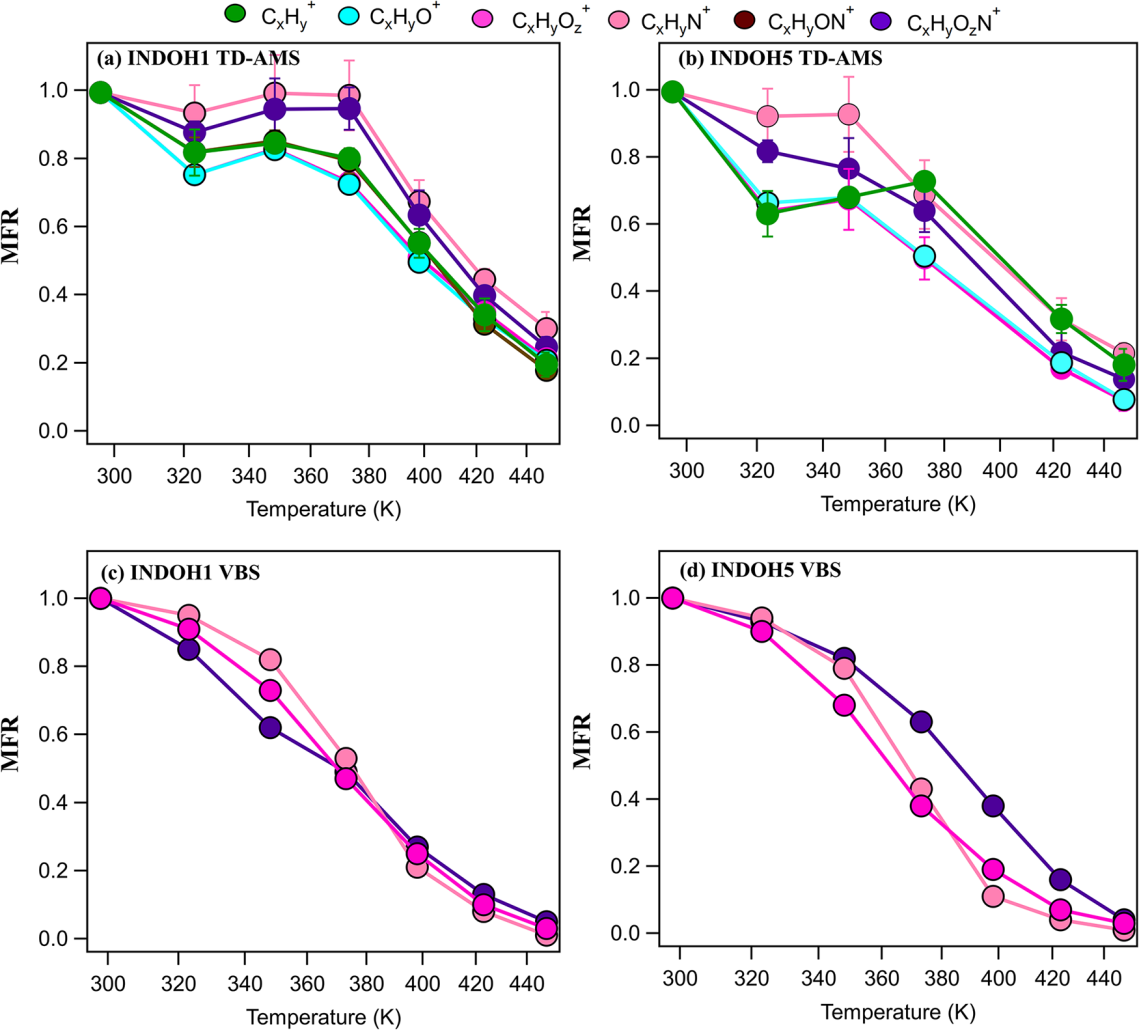
Mass fraction remaining (MFR) of indole SOA as measured by AMS for (a) 1-day and (b) 5-day OH-equivalent aged indole SOA, and as estimated from the VBS model for (c) 1-day and (d) 5-days OH-equivalent aged indole SOA samples. The colors represents the major chemical families, such as C_*x*_H_*y*_^+^, C_*x*_H_*y*_O^+^, C_*x*_H_*y*_O_*z*_^+^, C_*x*_H_*y*_N^+^, C_*x*_H_*y*_O_*z*_N^+^, and C_*x*_H_*y*_ON^+^. The MFR for each chemical family is calculated as the ratio of the mass concentration at the thermodenuder temperature to the corresponding mass concentration at room temperature.

For INDOH1 (Figure 5a), a clear volatility gradient is observed among the chemical families. Lightly oxygenated ions C_*x*_H_*y*_O^+^ exhibit the highest volatility, with *T*_50_ occurring around 390 K. The C_*x*_H_*y*_ family shows a similar but slightly lower thermal stability (*T*_50_ ≈ 395 K), followed by more oxygenated organic fragments (C_*x*_H_*y*_O_*z*_^+^) with intermediate volatility (*T*_50_ ≈ 400 K). In contrast, nitrogen-containing species show significantly lower volatility. The C_*x*_H_*y*_N ions have *T*_50_ around 410 K, while C_*x*_H_*y*_ON^+^ and C_*x*_H_*y*_O_*z*_N^+^ both exhibit the highest thermal stability, with *T*_50_ values near 415 and 410 K, respectively. These trends are generally consistent with the modeled thermograms in Figure 5c, and a similar volatility ranking is captured by the VBS among the chemical families. However, the VBS-derived *T*_50_ values are lower than those in AMS measurements, with C_*x*_H_*y*_O_*z*_^+^ and C_*x*_H_*y*_O_*z*_N^+^ near 370 K, whereas C_*x*_H_*y*_N^+^ showed *T*_50_ ∼ 377 K. *T*_50_ values in the VBS model are obtained by evaluating the temperature-dependent particle-phase fraction of each component, normalized to its initial condensed-phase abundance at 298 K. The VBS-derived *T*_50_ value corresponds to the temperature at which the normalized fraction falls to 0.5. In both indole systems, VBS-based MFR values at 448 K are consistently lower than those obtained from TD-AMS-based measurements, which can be attributed to multiple factors. For example, constituent vapors downstream of the TD may recondense onto particles, which could be the reason for the slight increase in MFR between 320 and 380 K (Figure 5a,b), thereby artificially raising MFR values, whereas the VBS model solely assumes that vaporized constituents never recondense onto particles.

Particle-phase viscosity from TD-AMS sampled aerosols also decreases molecular diffusion and further limits constituent evaporation rates, thereby increasing MFR values further. By contrast, the VBS model does not account for viscosity or assumes uniform viscosity at all temperatures for a modeled system, an assumption that is unlikely to hold true. The inverse correlation between viscosity and volatility,^72^ suggests that the viscosity of compound classes can influence VBS distributions. Particle-phase peak heights of more viscous N-containing species, for example, may shift toward less-volatile bins [log_10_(*C*_T_*) < 0], whereas less viscous components may enhance peak heights associated with more volatile bins [log_10_(*C*_T_*) > 0]. Overall, the extended retention of mass with heating for N-containing ions supports the inference that these species form the low-volatility core of the indole SOA under fresher aging conditions, consistent with observations in BrC systems.^64^

The volatility trend of INDOH5 is similar to that of INDOH1 (Figure 5b,d); however, the entire thermogram shifts toward lower temperatures. The *T*_50_ values from TD-AMS for C_*x*_H_*y*_^+^ and C_*x*_H_*y*_O^+^ decrease to ∼380 and ∼375 K, respectively, while C_*x*_H_*y*_O_*z*_^+^ exhibits a *T*_50_ of approximately 385 K. The nitrogen-containing ions also show a modest drop in thermal stability, with *T*_50_ around 395 K for both C_*x*_H_*y*_N^+^ and C_*x*_H_*y*_O_*z*_N^+^, and slightly higher at ∼400 K for C_*x*_H_*y*_ON^+^. In the VBS-derived thermograms (Figure 5d), C_*x*_H_*y*_O_*z*_^+^ shows a lower *T*_50_ of 364 K while C_*x*_H_*y*_N^+^ and C_*x*_H_*y*_O_*z*_N^+^ show *T*_50_ of 369 and 384 K, respectively. As stated above, the VBS models are governed by the Clausius–Clapeyron equation, which characteristically presumes that higher temperatures drive condensed-phase mass loss. This model only considers the gas-particle partitioning behavior of intact chemical molecules, whereas the TD-AMS apparatus can measure a slight enrichment of C_*x*_H_*y*_^+^, C_*x*_H_*y*_O^+^, and C_*x*_H_*y*_O_*z*_^+^ species in the 323–348 K range (Figure 5b) that are fragments of larger components, thereby artificially extending ion fragment-based *T*_50_ values. Furthermore, the Clausius–Clapeyron equation calculates equilibrium vapor pressure at each temperature, which may also explain why measured MFR values (panels a and b) are typically higher than modeled MFR values (panels c and d) due to kinetic limitation influences in the TD. Additionally, a lower initial tOM concentration of 10 μg/m^3^ for INDOH5 in the VBS distributions undergoes a greater percentage-based depletion rate of the particle-phase components compared to the initial 20 μg/m^3^ tOM specified for INDOH1. C_*x*_H_*y*_O_*z*_N^+^ constituent molecules undergo a slower particle-phase mass loss rate in INDOH5 in contrast to the INDOH1 system, potentially because after extensive aging, residual C_*x*_H_*y*_O_*z*_N^+^ components in the INDOH5 system are more chemically inert and less volatile compared to their INDOH1 counterparts.

There is no practical quantitative metric to describe the degree of overlap between AMS- and ESI­(+)-detected species used to construct the Figure 5 plots, since the former detects ion fragments, while the latter detects parent analyte ions. Therefore, the numerical values between the AMS and VBS-based thermograms are not directly comparable; the general volatility trends and relative changes with aging are well captured by the model. In INDOH1, the MFR behavior at lower temperatures (<360 K) shows good agreement between measured and modeled data, reflecting similar evaporation patterns for the more volatile components.

### 3.5 Optical Evolution with Chemical Aging and Thermal Processing

#### 3.5.1 Photochemical Evolution of Chromophore Components in Indole SOA

The PDA heatmap (photodiode array detection) for the 1-day OH aged sample (Figure 6a) displays multiple strong absorption features between 300 and 450 nm, with intense peaks particularly around RT = 4–5 and 11–12 min. These signals correspond to UV-absorbing chromophores, likely associated with conjugated indole oxidation products. In contrast, the INDOH5 heatmap (Figure 6b) shows a significant reduction in both peak intensity and spectral spread, suggesting degradation, fragmentation, or transformation of light-absorbing molecules due to prolonged OH exposure. The MAC­(λ) spectra (Figure 6c) further confirm this trend. The MAC values for INDOH1 reach up to ∼0.85 m^2^ g^–1^ at 300 nm, while those for INDOH5 drop to ∼0.35 m^2^ g^–1^, indicating an approximately 2-fold decrease in total absorbance. The VBS distributions resolved by AI_mod_ bins align with these optical measurements (Figure 4c,d). The particle-phase mass fraction of compounds with AI_mod_ < 0.5—indicative of nonaromatic compounds—increases from 83 to 91% from the INDOH1 to the INDOH5 sample. While the spectral shapes of both samples remain similar, exhibiting a decline in absorption toward 450 nm, the magnitude of absorption is consistently lower for the more aged SOA.

**Figure 6:**
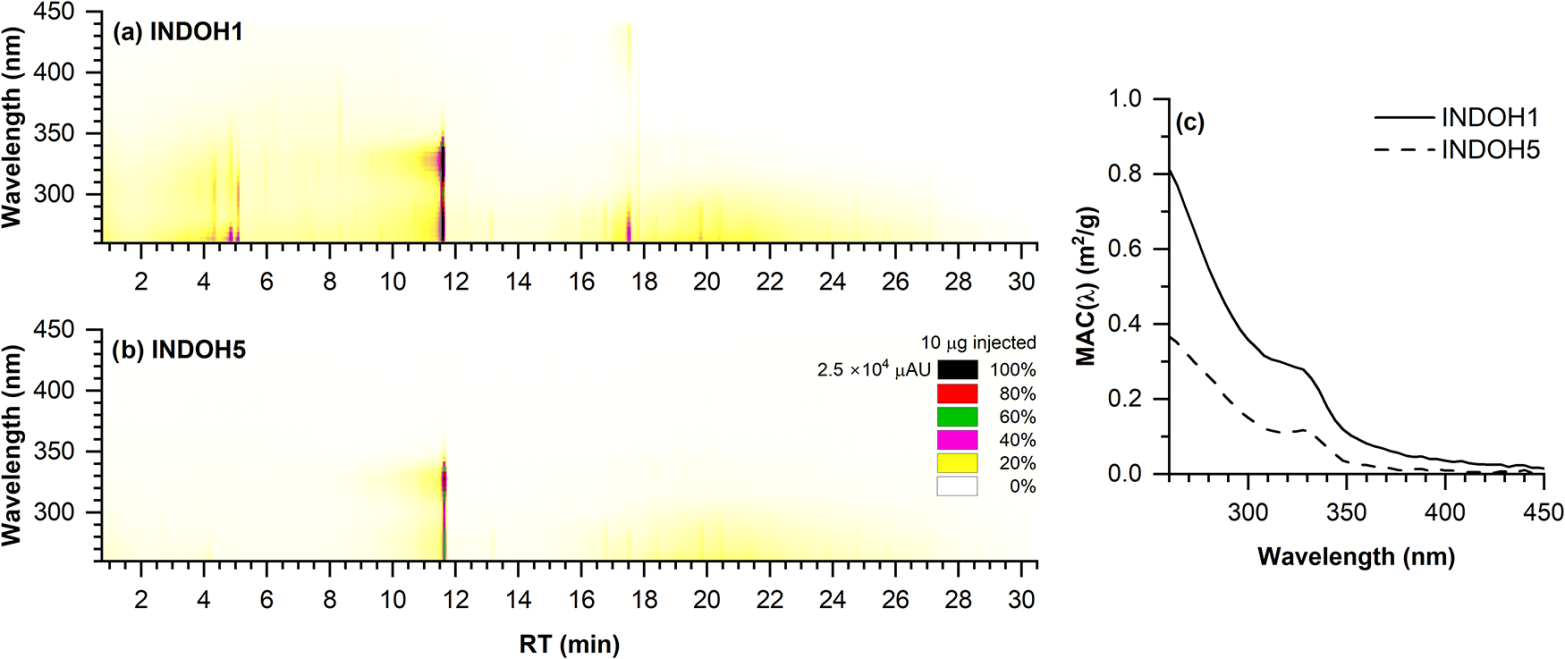
UPLC-PDA chromatograms of (a) INDOH1 and (b) INDOH5 samples and their corresponding (c) total MAC­(λ) plots. Chromatograms are color-coded by the relative absorption intensity, as denoted by the legend. Multiple light-absorbing features in the INDOH1 sample are consumed during prolonged OH aging and are absent in the INDOH5 sample, coupled with an absorbance decay by a factor of ∼2 from the INDOH1 to the INDOH5 sample.

The decrease in MAC­(λ) with aging reflects the transformation of light-absorbing compounds, likely driven by continued OH oxidation. A similar trend was reported by Montoya-Aguilera et al.^43^ and Jiang et al.,^70^ where chromophores such as isatin and tryptanthrin were shown to degrade into less absorbing species upon oxidation. These results support the susceptibility of nitrogen-containing chromophores in indole SOA to photochemical bleaching during atmospheric aging.^25^

#### 3.5.2 Temperature-Dependent Evolution of the Complex Refractive Index

The real and imaginary parts of the refractive index (RI) for indole SOA were determined as functions of TD temperatures using the methodology outlined in Section 2.3. The effect of heating on the complex RI of indole SOA is presented in Figure 7. At room temperature, the retrieved imaginary RI values were 0.014 ± 0.001 for INDOH1 and 0.005 ± 0.001 for INDOH5. A distinct dependence of the imaginary RI on TD temperature was observed, with maximum values of *k* ∼ 0.09 and 0.06 for INDOH1 and INDOH5, respectively, at a TD temperature of 448 K. The real part of RI retrieved for the room temperature is 1.549 ± 0.007 and 1.510 ± 0.002 for INDOH1 and INDOH5, respectively. An increase in the value of *n* is observed upon heating for INDOH5, with *n* changing from 1.50 to 1.55 as the temperature increases from 296.15 to 373.15 K. In contrast, for INDOH1, *n* remains nearly constant (∼1.55 to 1.57) up to 423.15 K, followed by a sudden increase (*n* reaching 1.60) upon further heating to 448.15 K. Previously, Kim and Paulson^73^ reported the impact of heating with a TD on the real part (*n*) of SOA generated from photooxidation and ozonolysis of limonene, α-pinene, and toluene. In their study, the *n* of α-pinene SOA with OH aging and heating (TD temperature of 338.15–358.15 K) was slightly higher (*n* ∼ 1.49–1.55) than *n* without heating (*n* ∼ 1.48–1.5). However, the *n* value for toluene SOA was unaffected by heating.

**Figure 7:**
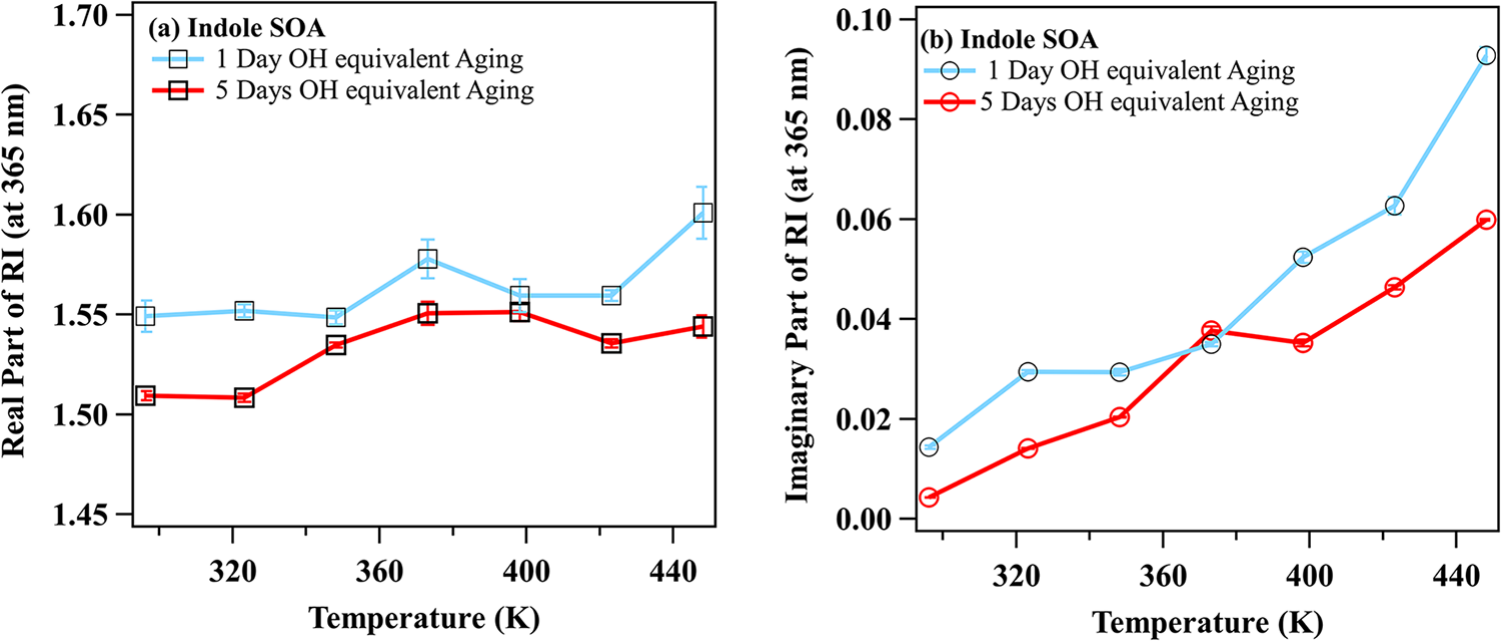
Variation of the retrieved (a) real and (b) imaginary parts of the refractive index (RI) at 365 nm with thermodenuder temperature for indole SOA. Cyan and red lines represent 1-day and 5-days equivalent aging, respectively. The RI of 100 nm indole SOA at each thermodenuder temperature was derived using inverse Mie theory based on the corresponding optical coefficients and number size distribution measurements.

Figure 7b shows the dependence of the imaginary part (*k*) of the RI on the temperature of the TD. At room temperature, the *k* values were 0.014 ± 0.001 for INDOH1 and 0.005 ± 0.001 for INDOH5. A distinct dependence of the *k* on TD temperature was observed, with maximum values of *k* ∼ 0.09 and 0.06 for INDOH1 and INDOH5, respectively, at a TD temperature of 448 K. The strong dependence of the *k* on the TD temperature suggests that the evaporating species are less absorbing, and as these volatile components are removed from the indole SOA upon heating, the residual aerosol phase becomes increasingly more absorbing.

To understand how these volatility-driven compositional changes influence optical properties, we examined the aromaticity of the detected species using the modified aromaticity index (AI_mod_). Figure 4c,d presents VBS distributions resolved by AI_mod_, highlighting the role of π-conjugated structures in governing light-absorbing behavior. In INDOH1, species with AI_mod_ > 0.67, indicative of condensed aromatic conjugation contribute trace levels of the mass. This fraction drops to less than 1% in INDOH5, pointing to significant chemical aging and fragmentation of π-conjugated structures. This compositional change mirrors the decline in chromophore content observed in the PDA chromatograms (Figure 6a,b) and the total MAC­(λ) spectra (Figure 6c), where INDOH5 exhibits nearly a 2-fold reduction in light absorption compared to INDOH1. The loss of π-conjugated, light-absorbing species with aging also manifests in the behavior of the temperature-dependent complex RI (Figure 7). INDOH1 shows a more pronounced increase in both the real (*n*) and imaginary (*k*) parts of the refractive index with heating, peaking at *n* ≈ 1.60 and *k* ≈ 0.09 at 448 K. In contrast, INDOH5 exhibits a more gradual change, with *k* remaining significantly lower (maximum ∼ 0.06), consistent with the absence of strongly absorbing chromophores. The temperature-dependent enhancement in *k* can be attributed to the selective evaporation of more volatile, weakly absorbing constituents during thermal processing, which leads to an enrichment of the aerosol phase in less volatile, strongly absorbing chromophores. These evaporated species likely correspond to volatile low-molecular weight (<250 g/mol) oxygenated monoaromatic compounds with phenolic moieties^32^ that were trapped onto the filter substrates during aerosol collection. At this high temperature of 448 K, the relatively less-oxygenated INDOH1 components—compared to the more oxidatively aged INDOH5 system—degas from the INDOH1 aerosols, leading to the higher *k* enhancement associated with the INDOH1 system. By contrast, the less volatile, more oxygenated INDOH5 system undergoes a less pronounced *k* enrichment, indicating that only a few components degas from the system, while those that remain continue to absorb light. We call this process darkening-by-volatilization, and it has been previously reported by Calderon-Arrieta et al.^32^ Together, Figures 4–7 demonstrate that the evaporation and OH bleaching lead to competing effects in the optical and chemical properties of indole SOA. Changes in volatility, functional group composition, and aromaticity directly govern the thermal and optical properties of indole SOA.

## 4 Atmospheric Implications

The thermodenuder (TD) experiments employed in this study provide a controlled framework to investigate volatility-driven compositional changes and their influence on optical properties. Heating in the TD accelerates the evaporation and repartitioning of semivolatile components, thereby mimicking the gradual gas–particle redistribution that occurs under atmospherically relevant conditions. This study reveals that the photochemical aging of SOA derived from indole significantly alters its chemical and optical properties, contributing significant insights into the atmospheric lifecycle of nitrogen-containing BrC aerosols. OH-induced oxidative aging leads to the degradation of low-volatility, nitrogen-containing chromophores, primarily π-conjugated CHON species, resulting in substantial photobleaching and increased overall volatility. These transformations suggest that the BrC light-absorbing ability diminishes over time under typical atmospheric oxidative conditions.

Simultaneously, a competing mechanism, darkening-by-volatilization, emerges, where the selective loss of weakly absorbing, more volatile components enriches the aerosol phase with more absorptive, lower-volatility species. This redistribution results in an absorbing and low-volatile core, supporting similar darkening behavior observed in previous studies.^32,74^

In the present study, OH-induced aging reduced light absorption by approximately a factor of 2, as the MAC (300 nm) decreased from ∼0.85 m^2^ g^–1^ (INDOH1) to ∼0.35 m^2^ g^–1^ (INDOH5) (Figure 6), indicating substantial photobleaching of nitrogen-containing chromophores. In contrast, heating in the thermodenuder caused a 4–6-fold enhancement in the imaginary refractive index (*k*), increasing from 0.014 to 0.09 for INDOH1 and from 0.005 to 0.06 for INDOH5 (Figure 7). The magnitude of this enhancement in *k* is consistent with, though somewhat higher than, the absorption increases reported for other BrC systems, most of which are expressed in terms of the MAC rather than *k*. Calderon-Arrieta et al.^32^ showed that the removal of volatile organics from biomass-burning tar increased the MAC (405 nm) from 0.1 to 0.5 m^2^ g^–1^, corresponding to a 5-fold increase in absorption. Zhou et al.^75^ showed that evaporation induced transformations in Volatile Chemical Products-derived SOA increased MAC by ∼4 fold at 280 nm (∼3.7 folds at 400 nm), causing pronounced browning. They attributed this to peroxide decay and the formation of highly conjugated N-containing chromophores during evaporation, with the effect peaking near ∼40% RH. Fang et al.^76^ reported that *k* (at 300 nm) for SOA produced from secondarily evaporated biomass-burning vapors remains almost 0.016–0.017 after OH aging from 0.7 to 5.5 days. Al-Mashala et al.^77^ reported that UV irradiation enhanced both the light absorption and viscosity of primary biomass-burning BrC, with wavelength-dependent increases in MAC up to ∼70% at 445 nm.

Taken together, these studies indicate that volatilization or evaporation generally enhances BrC absorption, with the extent varying across systems and wavelengths. The enhancement observed in the present study (up to 6-fold in *k*) lies within or slightly above the upper range of previously reported effects, underscoring the quantitative significance and atmospheric relevance of the darkening-by-volatilization mechanism identified here. The conclusions of this study assume that the dominant effect of thermodenuder heating is physical volatilization. While no direct evidence for thermally induced chemical transformations or the formation of new light-absorbing compounds was detected in the present experiments or represented in the VBS modeling, the possibility that such processes may occur under elevated temperatures cannot be fully excluded and is therefore acknowledged as a limitation of the experimental approach.

While this study demonstrates a clear link between chemical aging and optical changes in indole SOA, further investigations on a broader range of BrC aerosols are needed. In particular, studies that combine volatility, molecular structure, optical properties, and aging pathways across diverse aromatic and nitrogen-containing compounds will be essential to constrain the lifecycle of BrC aerosols in the atmosphere. Extending this framework to other major BrC sources, such as biomass-burning-derived aerosols^32^ or other types of SOA, could reveal analogous darkening-by-volatilization processes with important implications for atmospheric radiative effects and their representation in climate models.

## Supplementary Material



## Notes

Notes on aerosol optical properties and retrieval of complex refractive index; offline chemical analysis; UPLC-PDA-HRMS instrument parameters for offline chemical analysis; optical measurements of filter samples; molecular characterization; saturation mass concentration and enthalpy of vaporization calculations for indole SOA VBS distributions; proposed OH-induced reaction mechanism of photooxidation of indole; and gas-particle partitioning trends in INDOH1 and INDOH5 VBS distributions resolved by compound class. Figures of ESI­(+) mass spectra for indole SOA samples; UpSet graph displaying common and unique masses present; TICs of samples acquired in ESI­(+) mode; HR-ToF-AMS mass spectra of indole SOA generated from the OH oxidation; assigned peaks and 20 most intense peaks in indole SOA samples acquired in ESI­(+) mode: ESI­(+) mass spectrum for indole SOA with peaks identified; and temperature-resolved VBS distributions of indole SOA samples, resolved by compound class (C_*x*_H_*y*_O_*z*_, C_*x*_H_*y*_N_1≤_, C_*x*_H_*y*_O_*z*_N_1≤_) across temperatures from 298 to 373 K (PDF)

## ⊥

Department of Atmospheric Sciences, Texas A&M University, College Station, Texas 77843, United States
