# Supplementary material for: Photochemical Aging of Indole SOA: Implications for Volatility and Optical Properties
Source: Environ Sci Technol. 2026 Feb 24;60(9):7237–50. doi: 10.1021/acs.est.5c10237 (PMC12980833; doi:10.1021/acs.est.5c10237)
Supplement: Supplementary file 1 [file es5c10237_si_001.pdf]

Supplementary Information for

Photochemical Aging of Indole SOA: Implications for Volatility and Optical Properties

Thenoor Chandran Ajith[1#], Diego Calderon-Arrieta[2], Hongwei Pang[1], Zheng Fang[1], JingKai Wang[2], Jessica Knull[2], Nyiri Hajian[2], Kirby Hill[2], Chunlin Li[3], Alexander Laskin[2,4], and Yinon Rudich[1*]

1Department of Earth and Planetary Sciences, Weizmann Institute of Science, Rehovot, 7610001, Israel

2Department of Chemistry, Purdue University, West Lafayette, IN 47907, United States

3College of Environmental Science and Engineering, Tongji University, Shanghai, 200092, China

4Department of Earth, Atmospheric and Planetary Sciences, Purdue University, West Lafayette, IN 47907, United States.

Presently at the Department of Atmospheric Sciences, Texas A&M University, College Station, Texas, 77843, United States

***Corresponding author**: Yinon Rudich (yinon.rudich@weizmann.ac.il)

Table of Contents

Supplemental Note A. Aerosol optical properties and retrieval of complex refractive index

Supplemental Note B. Offline chemical analysis

Supplemental Note C. *UPLC-PDA-HRMS instrument parameters for offline chemical analysis*

Supplemental Note D. Optical measurements of filter samples

Supplemental Note E. Molecular characterization

Supplemental Note F. *Saturation mass concentration and enthalpy of vaporization calculations for indole SOA VBS distributions.*

Supplemental Note G. Proposed OH induced reaction mechanism of photooxidation of indole

Supplemental Note H. *Gas-particle partitioning trends in INDOH1 and INDOH5 VBS distributions resolved by compound class*

Supplementary Figure S1: ESI(+) Mass Spectra for (a) INDOH1 and (b) INDOH5 samples

Supplementary Figure S2: Upset graph displaying common and unique masses present in INDOH1 and INDOH5 samples.

Supplementary Figure S3: TICs of (a) INDOH1 and (b) INDOH5 samples acquired in ESI(+) mode.

Supplementary Figure S4: HR ToF AMS mass spectra of Indole SOA generated from the OH oxidation with an equivalent aging of 1 day (INDOH1).

Supplementary Figure S5: HR ToF AMS mass spectra of Indole SOA generated from the OH oxidation with an equivalent aging of 5 days (INDOH5).

Supplementary Figure S6: Assigned and 20 most intense peaks in (a) INDOH1 and (b) INDOH5 samples acquired in ESI(+) mode. The top 20 most intense peaks are color-coded based on AI_{mod} category, while the other assigned peaks are color-coded based on compound class.

Supplementary Figure S7. ESI (+) mass spectrum for (a) INDOHI and (b) INDOHI5 with peaks identified

Supplementary Figure S8. Scheme 1- Proposed mechanism for the reaction of indole with OH

Supplementary Figure S9: Temperature-resolved VBS distributions of (a) INDOH1 and (b) INDOH5 samples, resolved by compound class ($C_xH_yO_z$, $C_xH_yN_{1\leq}$, $C_xH_yO_zN_{1\leq}$) across temperatures from 298 to 373 K. OM values are maintained at 10 μg/m^3 and 20 μg/m^3 for INDOH1 and INDOH5, respectively. Pie graphs display the total gas-phase and particle-phase abundances of the four compound classes, and background colors denote the five VOC bins. INDOH1 undergoes a more rapid loss of particle-phase CHON compounds than INDOH5 does, which highlights the more chemically inert INDOH5 aged constituents.

Supplemental Note A. Aerosol optical properties and retrieval of complex refractive index

A newly designed SSA monitor at 365 nm (described in detail by Ajith et al. [1]) was used to measure the optical properties of the indole SOA. The SSA monitor provides real-time scattering and extinction coefficients at a wavelength of 365 nm. The working principle of the SSA monitor used in this study is similar to that of the previous SSA monitor versions and described in detail by Onasch et al.[2] and Modini et al.[3]. Briefly, extinction measurements are performed in an optical cavity with an LED light source, highly reflective mirrors (Reflectivity ~0.9998; FiveNine Optics), and a vacuum photodiode detector. Aerosol samples enter the cavity at 0.85 L/min, where a square-wave modulated LED beam interacts with them, causing phase shifts. These shifts, linked to the optical properties of aerosols, are used to determine extinction coefficients[2]. The instrument determines scattering coefficients using an integrating sphere surrounding the optical cavity. The integrating sphere is coated with Avian D white reflectance coating (Reflectivity ~94% at 365 nm, Avian Technologies, http://aviantechnologies.com). The light from all directions except extreme forward and backward directions is reflected inside the integrating sphere and focused on a PMT (photomultiplier tube) mounted inside. The instrument was calibrated following the approach

outlined by Modini et al.[3]. The study used 100 nm particles, which do not require truncation corrections, so no such corrections were applied.

The complex refractive index (RI) of indole SOA was determined from the scattering coefficients and absorption coefficients (derived via the EMS method[3]) measured by the SSA monitor along with SMPS measurements. The retrieval process used the open-source Python package *PyMiescatt*, utilizing its contour intersection approach. This method calculates scattering and absorption coefficients for a given size distribution, wavelength, and a range of RI values (n and k). The measured coefficients, including their standard deviations, are mapped onto contour plots in n-k space, where their intersection points provide the retrieved RI values. Further details on *PyMiescatt* are on its developer's website (https://pymiescatt.readthedocs.io/en/latest/) and related publications[4–10]. The retrieval of the complex RI using this approach was validated by using standards of polystyrene nanospheres (representing purely scattering aerosols) and nigrosin (representing absorbing aerosols). Particles with mobility diameters of 105 nm for polystyrene nanospheres and 150 nm for nigrosin were generated using this setup. Even though polystyrene nanospheres are monodispersed, we used the AAC to further size selection to avoid unwanted clustering of these particles in the other sizes. The retrieved real and imaginary parts of RI for polystyrene nanospheres (n=1.652±0.004, k=0) and nigrosin (n=1.690±0.010, k=0.131±0.003) agree with the reported values of polystyrene latex spheres (n=1.651 by Washenfelder et al.[11] at wavelength =360 nm) and nigrosin (n=1.676±0.008 and k=0.147±0.008 by Bluvshtein et al.[12] at wavelength =365 nm).

Supplemental Note B. Offline chemical analysis

Quartz fiber filters positioned downstream of the room temperature-programmed TD were used to collect the indole-SOA samples for offline optical and chemical measurements. The filters were placed in Petri dishes, sealed with paraffin film, and stored at –80 °C until shipment at Weizmann Institute of Science, Israel. All offline analyses were performed at Purdue University, West Lafayette, USA. For transportation, the sealed filters were packed in a thermally insulated container with dry ice to maintain sub-zero temperatures and the filters were confirmed to be in good condition upon receiving. Quarter sections of a blank filter, a sample filter containing INDOH1 materials, and a sample filter containing INDOH5 materials were cut, and deposited analytes on each filter were dissolved in three separate scintillation vials containing 3 mL of acetonitrile (ACN, Optima LC/MS grade, Fisher Chemical). ACN was

selected as the extraction solvent due to its proven efficacy in extracting BrC constituents[13,14], its compatibility with the LC separation protocol employed[14,15], and inertness with the analytes[16,17]. Sonication for 20 minutes was employed to enhance indole-SOA extraction efficiency. Insoluble indole-SOA constituents were removed by filtering the extracts through PTFE membrane syringe filters (0.2 μm, Fisher brand) prewetted with ACN. To maximize recovery, the extraction vials were rinsed three times with 1 mL with ACN (Optima LC/MS grade, Fisher Chemical) and the rinsates were passed through the membrane filter. The combined extracts and rinsates were subsequently evaporated under a gentle stream of N_2 gas to concentrate the solutions to ~400 μg/mL mass concentration. More volatile components may have escaped during the evaporative preconcentration step, potentially biasing the datasets towards enrichment of less volatile SOA products. The preconcentrated filter extracts were analyzed using a hyphenated platform consisting of ultra high-performance liquid chromatography (UPLC), photodiode array detection (PDA), and high-resolution mass spectrometry (HRMS). 25 μL of the 400 μg/mL solutions were injected into a Vanquish HPLC system equipped with a Light Pipe flow cell and coupled to a high-resolution orbitrap mass spectrometer (Q Exactive HF-X, ThermoFisher) to achieve an injected mass of 10 μg per sample. Separation of chemical constituents was performed using a reversed-phase Luna Omega Polar C18 column (Phenomenex, 00B-4742-AN), maintained at room temperature[18]. A Security Guard ULTRA Cartridge (Phenomenex, AJ0-9505) was installed to prevent column contamination by insoluble particles. A binary mobile phase consisting of water (A) and ACN (B), both Optima LC/MS grade and containing 0.1% (v/v) formic acid, was used at a flow rate of 0.4 mL/min.[18] An electrospray ionization (ESI) source, operated in positive ion mode, was used to ionize the SOA constituents into the HRMS instrument.

Supplemental Note C. *UPLC-PDA-HRMS instrument parameters for offline chemical analysis*

The gradient elution method occurred as follows: 0−0.5 min at 5% B, 0.5−26 min a linear ramp up to 100% B, 26−30.5 min held at 100% B, and 31.0-36.0 min restored to 5% B to re-equilibrate the column for the next sample[18]. Separated constituents flowed into the PDA detector before eluting into the HRMS. The PDA detector took scans at a 20 Hz rate and recorded 200−680 nm absorbance with a 2 nm spectral resolution. Optical and chemical properties of blank filter extracts with ACN were measured to remove interferences attributed to the filter, extraction solvent, and mobile phase. Electrospray ionization (ESI) in positive ion mode was used to ionize the SOA constituents. The ESI source was programmed with the

following settings: 300 °C capillary temperature, 250 °C probe temperature, 3.5 kV spray voltage, 50 arbitrary units of sheath gas, 12 arbitrary units of auxiliary gas, and 2.5 arbitrary units of sweep gas. Mass spectra were acquired with an 80−1200 m/z range and mass resolution 240,000 at 200 m/z. Mass calibration in positive ion mode was conducted with a commercial solution (ThermoFisher, PI-88323).

Supplemental Note D. Optical measurements of filter samples

The UV light absorbance per unit of organic mass is defined as the mass absorption coefficient, MAC (λ)[19], which was calculated from the LC-PDA records using Equation S1[20]

$$MAC(\lambda)(m^2g^{-1}) = \frac{Abs(\lambda) \times \Delta t \times \ln(10) \times F}{b \times 10 \times m_{inj}} \qquad (S1)$$

where Abs(λ) (μAU) is the PDA-recorded wavelength-dependent absorbance integrated over the entire elution time range, Δt (0−30.5 min). 1 AU (Absorbance Unit) corresponds to a base-10 logarithmic absorbance of 1, meaning the transmitted light is reduced to one-tenth of the incident intensity. F (mL/min) is the mobile phase flow rate, b is the optical path length (1.0 cm), and m_{inj} (10000 ng) is the mass injected into the HPLC system. The combined unit conversions from ng to g, μAU to AU, mL to cm^3, cm to m, and cm^3 to m^3 are reflected by a factor of 10 in the denominator. It is assumed that all the injected mass is organic mass, and, therefore, this is an underestimation of the MAC(λ) attributed to organic mass present in the indole SOA samples. This is the case, because organic mass constitutes only a fraction of the entire injected mass. Consequently, the calculated m_{inj} value is overestimated, and using the organic component of m_{inj} as the denominator would yield a higher MAC(λ) value.

Supplemental Note E. Molecular characterization

MZMine 2.53, an open-source LC-MS data processing software (https://mzmine.github.io/), was used to extract ion chromatograms from the background-subtracted mass spectra.[21] Individual peaks were grouped based on homologous series using custom-built Excel macros,[22] and elemental constraints for formula assignment in ESI(+) mode were as follows: 0−∞ C, 0−∞ H, 0−5 N, 0−50 O, and 0−1 Na. Singly-charged $[M+H]^+$ and $[M+Na]^+$ ions with <500 m/z were considered, and elemental formulas were subsequently assigned using the MIDAS molecular formula calculator (v. 1.2.3; National High Magnetic Field Laboratory, USA). Peaks with formula assignments that deviated from the error clustering trend were considered erroneous, and their initial assignments were deleted. Accurately assigned species were categorized into

CHO, CHN, and CHON classes. The ESI (+) high-resolution mass spectra of the two analyzed samples are displayed in Figure S1. The Upset plot used to identify unique and common species in the two samples is conveyed in Figure S2. Total ion chromatograms (TICs) displaying separation of the samples are provided in Figure S3. Double bond equivalency[23] (DBE), saturation mass concentrations[24] (C^*_{298K}, μg/m^3) and modified aromaticity index[25] (AI_{mod}) were calculated from the elemental formulas of the corresponding neutral molecules. DBE indicates the degree of unsaturation in the molecule and is calculated based on the equation represented below.

$$DBE = C - \frac{H}{2} + \frac{N}{2} + 1 \qquad (S2)$$

where C, H, and N denote the quantity of carbon, hydrogen, and nitrogen elements in the assigned molecule. The AI_{mod} coefficient builds from the DBE calculation and indicates the conjugated π-bond network of assigned molecules. The AI_{mod} calculation is displayed in Equation S3 below.

$$AI_{mod} = \frac{DBE_{AI}}{C_{AI}} = \frac{1 + C - \frac{1}{2}O - \frac{1}{2}(N + H)}{C - \frac{1}{2}O - N} \qquad (S3)$$

where C, O, H, and N are numbers of carbon, oxygen, hydrogen, and nitrogen atoms in each of the individual species[25]. C_{AI} indicates the quantity of potential double bonds associated with O and N heteroatoms in the species. AI_{mod} is assumed to be 0 if either of the following conditions is met: $AI_{mod} < 0$ or $C_{AI} < 0$. AI_{mod} is restored to a value of 1 if $AI_{mod} > 1$[25]. Volatility values and enthalpies of transition of the detected species were calculated using a set of parameterization equations[26–28] summarized in Supplemental Note F. Using these values, representative VBS distributions of these indole SOA aged compounds were generated to visualize their composition in the gas and aerosol phases. After volatility bin heights were established for each indole-SOA system, particle-phase and gas-phase mass concentrations for assigned compounds were obtained by weighing their respective particle-phase and gas-phase mass fractional values to the volatility bin heights to which they correspond.

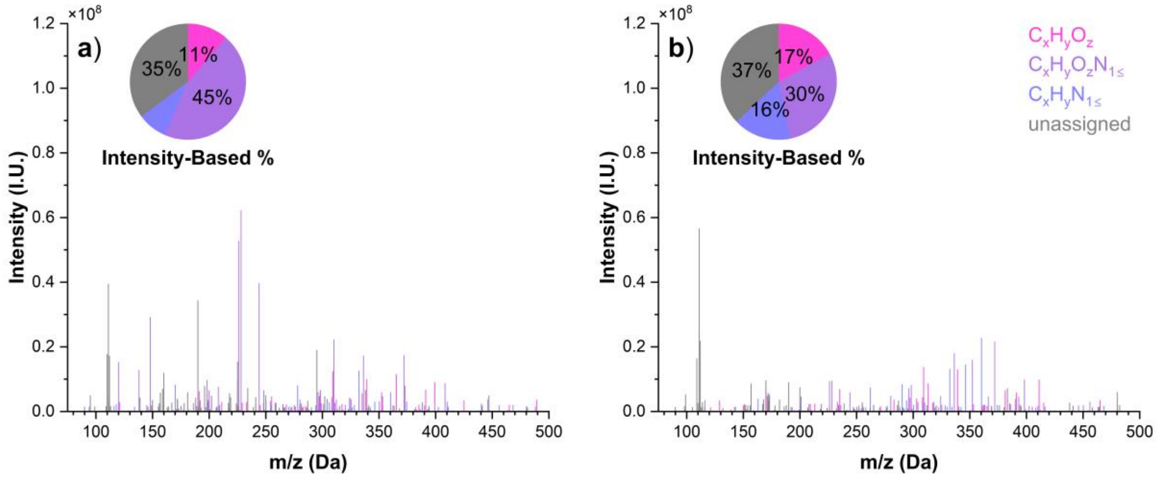

Supplementary Figure S1: ESI(+) Mass Spectra for (a) INDOH1 and (b) INDOH5 samples

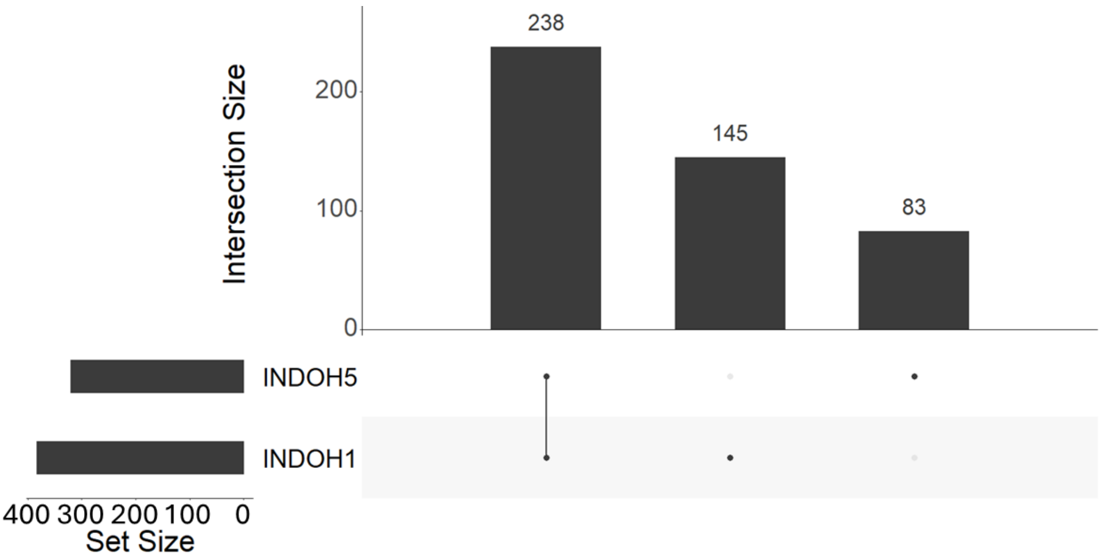

Supplementary Figure S2: Upset graph displaying common and unique masses present in INDOH1 and INDOH5 samples.

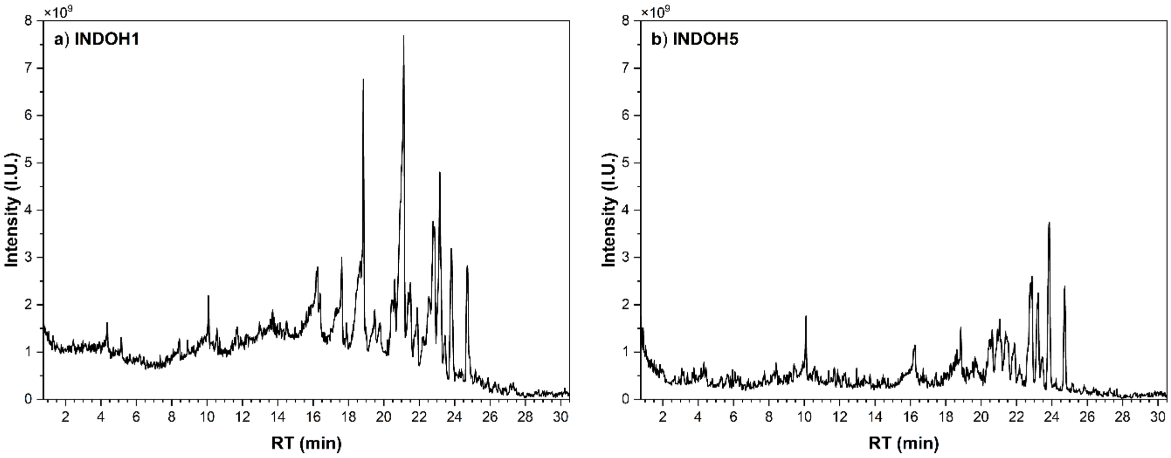

Supplementary Figure S3: TICs of (a) INDOH1 and (b) INDOH5 samples acquired in ESI(+) mode.

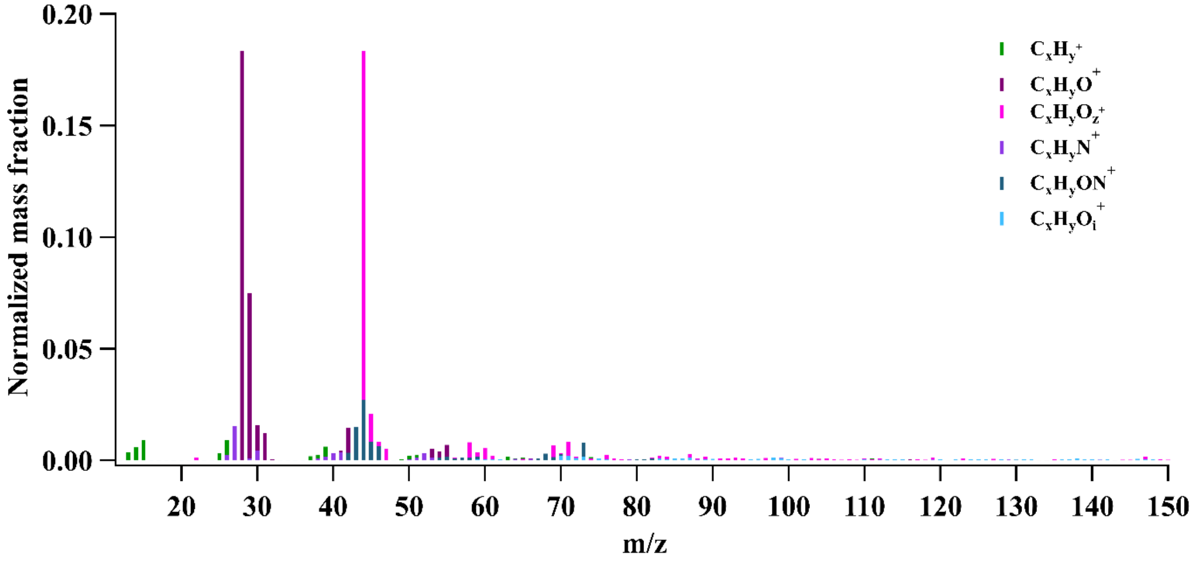

Supplementary Figure S4: HR ToF AMS mass spectra of Indole SOA generated from the OH oxidation with an equivalent aging of 1 day (INDOH1).

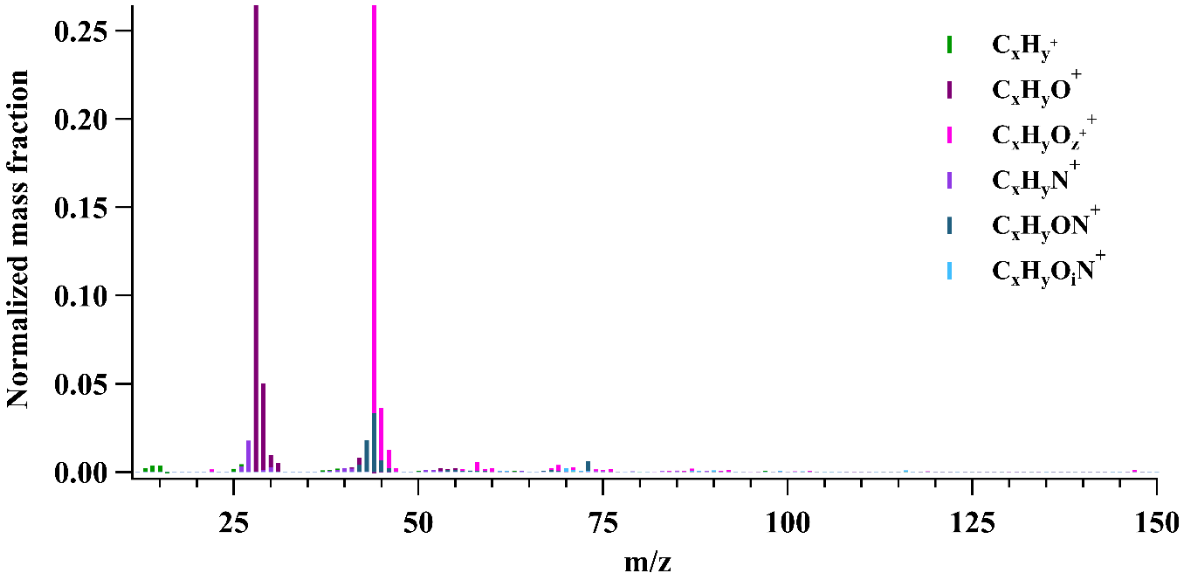

Supplementary Figure S5: HR ToF AMS mass spectra of Indole SOA generated from the OH oxidation with an equivalent aging of 5 days (INDOH5).

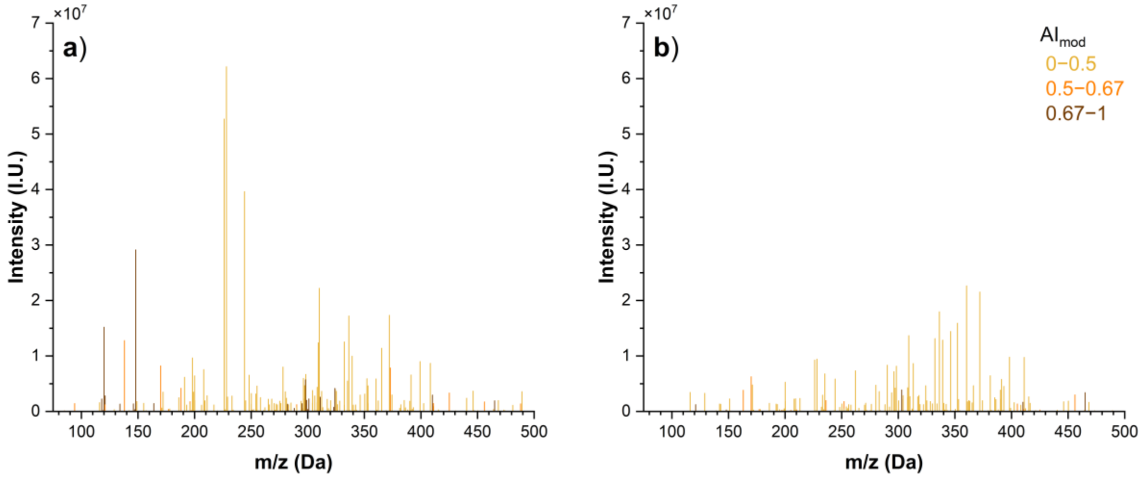

Supplementary Figure S6: ESI(+) high-resolution mass spectra for (a) INDOH1 and (b) INDOH5 samples. Assigned peaks are color-coded based on AI_{mod} category.

Supplemental Note F. *Saturation mass concentration and enthalpy of vaporization calculations for indole SOA VBS distributions.*

Expected saturation mass concentrations of indole SOA components for VBS distributions of the two systems involved a few sequences of calculations. First, saturation mass concentrations for assigned constituents were estimated based on the molecular corridor equation represented below[29].

$$\log_{10}\left(C^{0}_{298K},\frac{\mu g}{m^3}\right) = (n_C^0 - n_C)b_C - n_O b_O - 2\frac{n_C n_O}{n_C + n_O}b_{CO} - n_N b_N \qquad (S4)$$

where n_C, n_O, and n_N represent the counts of their corresponding elements; n^0_C corresponds to the reference carbon number; and the b coefficients are scaling factors. These coefficient values are reported in Table 1 of Li et al.[29] . Next, these values were adjusted based on a correlation equation developed in a previous work that also measured SOA products[26,30]

$$\log_{10}\left(C^{*}_{298\,K},\frac{\mu g}{m^3}\right) = 1.0491 \times \log_{10}\left(C^{0}_{298\,K},\frac{\mu g}{m^3}\right) - 1.8393 \qquad (S5)$$

To estimate the gas-particle partitioning behavior of assigned chemical compounds, the enthalpy of transition (ΔH^*) was derived for each compound using the equation featured in Ranjan et al.[31].

$$\Delta H^*\left(\frac{kJ}{mol}\right) = -11 \times \log_{10}\left(C^{*}_{298\,K},\frac{\mu g}{m^3}\right) + 85 \qquad (S6)$$

VBS is a framework that employs $C^*_{298\,K}$ and ΔH^* of organic aerosol compounds to visualize the gas-phase and aerosol-phase distributions of complex aerosol mixtures at specific total

organic mass (tOM) loadings and temperatures. Following protocols outlined in previous works[26,32,33]. VBS distributions for both INDOH1 and INDOH5 samples were generated with temperatures ranging between 298−373 K.

Supplemental Note G. Proposed OH induced reaction mechanism of photooxidation of indole

Based on the assigned peaks detected in the ESI (+) high-resolution mass spectrum shown in Figure S7 for INDOHI (a) and INDOHI5 (b), products were identified as 3-oxindole (133.053 g/mol), anthranilic acid (137.048 g/mol), isatin (147.032 g/mol) and isatoic anhydride (163.027 g/mol). As shown in Figure S8 scheme 1, the H-abstraction by OH from C=C double bond of pyrrole ring followed by reaction with O_2 and RO_2 leads to the formation of A1[34], A1 may proceed via two channels. One channel is to abstract hydrogen from another indole molecule to give 3-oxindole. In the second channel, A1 interacts with O2 and RO2 to generate isatin. Similarly, the H-abstraction reaction also results in the production of isatoic anhydride. Alternatively, the OH addition to pyrrole ring followed by interaction with O_2 and RO_2 yields alkoxy radical A2, which reacts with O2 to produce A3[35]. A3 undergoes H-abstraction by OH and subsequent decarbonylation forming A4, which can abstract H from another indole to give anthranilic acid[36].

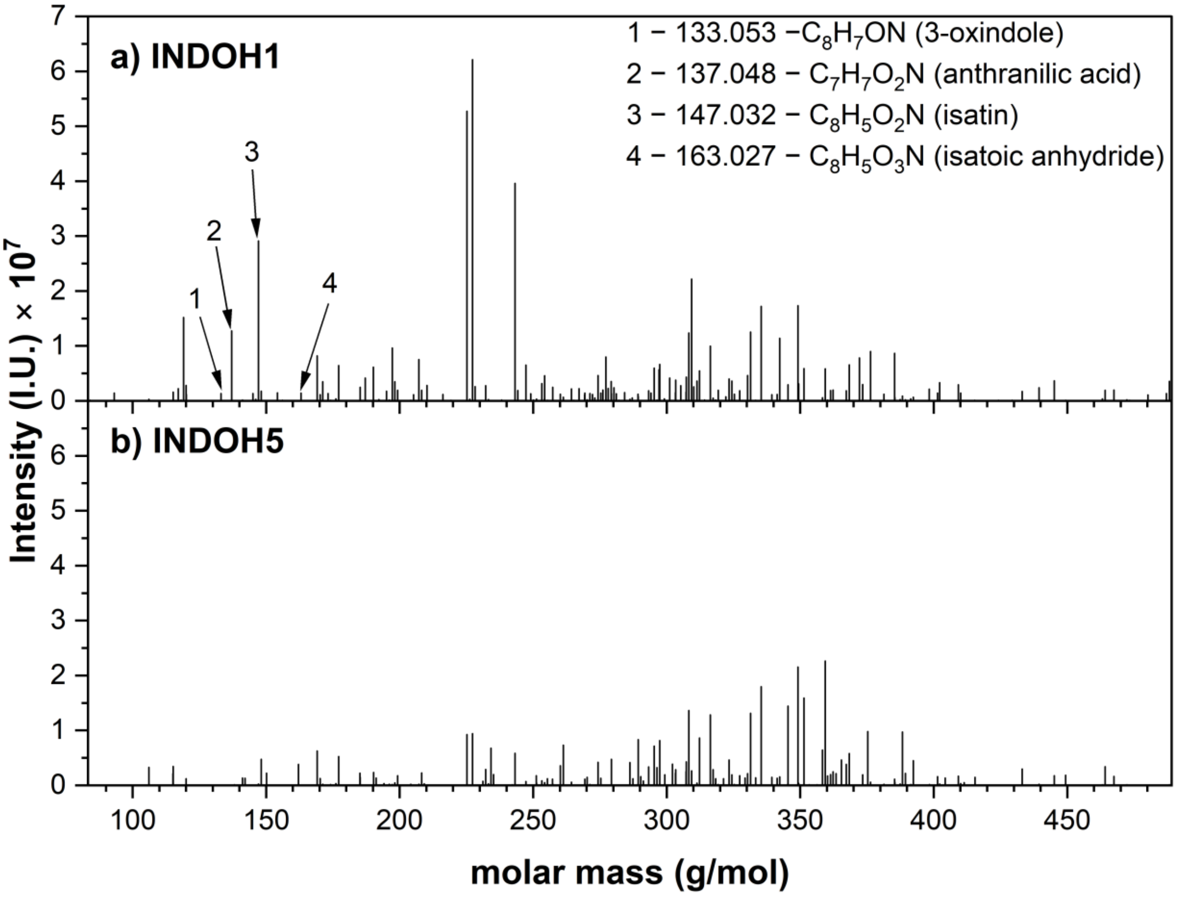

Figure S7. Assigned peaks in the ESI (+) high-resolution mass spectra for INDOHI (a) and INDOHI5 (b)

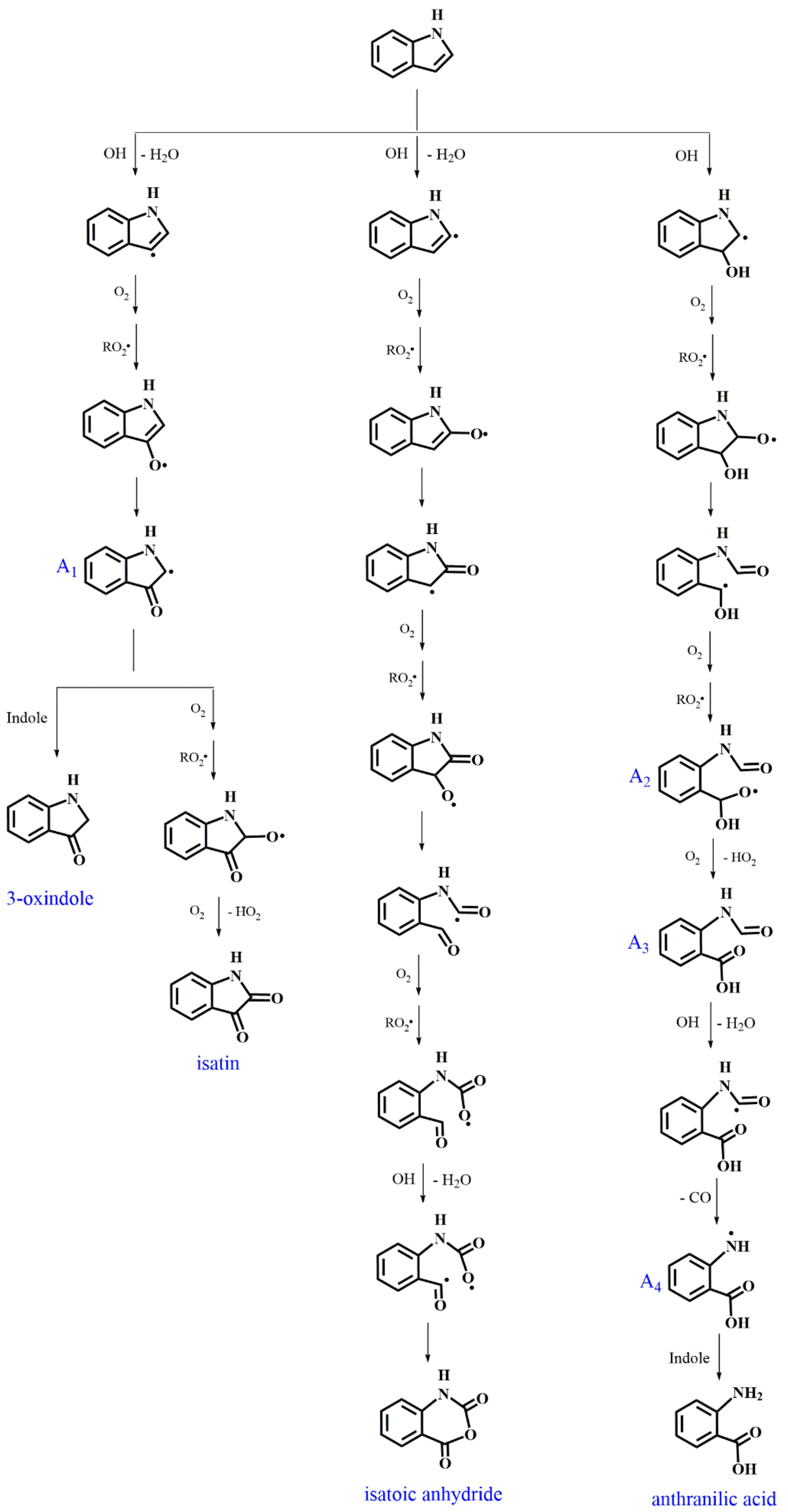

Figure S8. Scheme 1: Proposed mechanism for the reaction of indole with OH

Supplemental Note H. *Gas-particle partitioning trends in INDOH1 and INDOH5 VBS distributions resolved by compound class*

To visualize the anticipated gas-particle partitioning trends of INDOH1 and INDOH5, VBS distributions of the two samples with compound class resolution were generated with temperature values ranging from 298−373 K, and these graphs are displayed in Figure S9. In INDOH1 (Figure S5a), CHON compounds dominate the system (64% total particle-phase abundance) with more than 90% of the mass in the particle phase. As temperature increases, the CHON fraction drops to 31% at 373 K, and the overall particle-phase fraction decreases to less than 50%. Despite the CHON decay in overall particle abundance at 373 K, CHON compounds make up more than 50% of the total particle-phase abundance of the INDOH1 system. This consistent trend aligns with the AMS thermogram, where nitrogen-containing ions show higher thermal stability (T_{50} ~410−415 K) compared to the CH and CHO ions (~390−395 K), confirming that CHON species drive the low-volatility character of less aged SOA.

The VBS changes for the INDOH5 system as a function of temperature are conveyed in Figure S5b. INDOH5 exhibits a narrower volatility profile with CHON species dominating through (46% at 298 K). The particle phase declines from 95% at 298 K to less than 50% at 373 K, indicating similar volatility to the INDOH1 system. While INDOH1 and INDOH5 exhibit similar particle-phase mass fractions at 298 K and at 373 K, INDOH5 undergoes a slower particle phase mass loss than INDOH1 at the intermediate 323 and 348 K temperatures. This is due to the narrower volatility profile of INDOH5 distribution that features more nonvolatile compounds than the INDOH1 sample. While more chemically-inert SOA is present in INDOH5, the OH oxidative aging mechanism can still generate less aromatic and weaker chromophoric aged products[37].

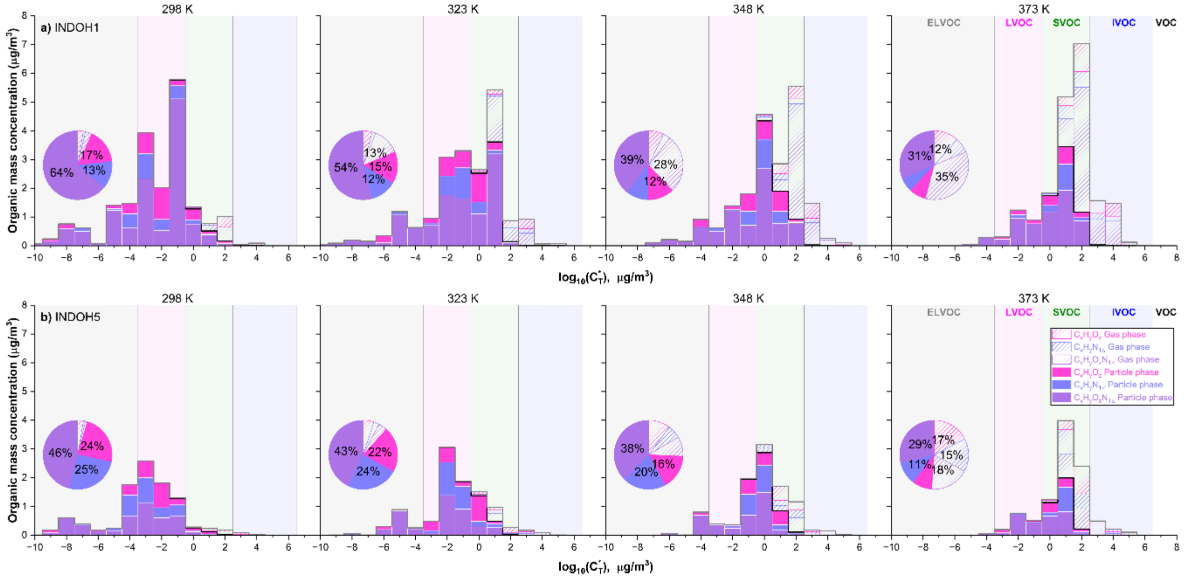

Supplementary Figure S9: Temperature-resolved VBS distributions of (a) INDOH1 and (b) INDOH5 samples, resolved by compound class ($C_xH_yO_z$, $C_xH_yN_{1\le}$, $C_xH_yO_zN_{1\le}$) across temperatures from 298 to 373 K. OM values are maintained at 10 µg/m^3 and 20 µg/m^3 for INDOH1 and INDOH5, respectively. Pie graphs display the total gas-phase and particle-phase abundances of the four compound classes, and background colors denote the five VOC bins. INDOH1 undergoes a more rapid loss of particle-phase CHON compounds than INDOH5 does, which highlights the more chemically inert INDOH5 aged constituents.

References

(1) Ajith, T. C.; Windwer, E.; Fang, Z.; Li, C.; Modini, R. L.; Onasch, T. B.; Freedman, A.; Rudich, Y. Evaluation of the 365 nm CAPS PM SSA Monitor and Its Use in Both Laboratory and Field Measurements. *Aerosol Sci. Technol.* **2025**, 1–19. https://doi.org/10.1080/02786826.2025.2488478.

(2) Onasch, T. B.; Massoli, P.; Kebabian, P. L.; Hills, F. B.; Bacon, F. W.; Freedman, A. Single Scattering Albedo Monitor for Airborne Particulates. *Aerosol Sci. Technol.* **2015**, *49* (4), 267–279. https://doi.org/10.1080/02786826.2015.1022248.

(3) Modini, R. L.; Corbin, J. C.; Brem, B. T.; Irwin, M.; Bertò, M.; Pileci, R. E.; Fetfatzis, P.; Eleftheriadis, K.; Henzing, B.; Moerman, M. M.; Liu, F.; Müller, T.; Gysel-Beer, M. Detailed Characterization of the CAPS Single-Scattering Albedo Monitor (CAPS PMssa) as a Field-Deployable Instrument for Measuring Aerosol Light Absorption with the Extinction-Minus-Scattering Method. *Atmospheric Meas. Tech.* **2021**, *14* (2), 819–851. https://doi.org/10.5194/amt-14-819-2021.

(4) Sumlin, B. J.; Heinson, W. R.; Chakrabarty, R. K. Retrieving the Aerosol Complex Refractive Index Using PyMieScatt: A Mie Computational Package with Visualization Capabilities. **2017**. https://doi.org/10.48550/ARXIV.1710.05288.

(5) Sumlin, B. J.; Pandey, A.; Walker, M. J.; Pattison, R. S.; Williams, B. J.; Chakrabarty, R. K. Atmospheric Photooxidation Diminishes Light Absorption by Primary Brown Carbon Aerosol from Biomass Burning. *Environ. Sci. Technol. Lett.* **2017**, *4* (12), 540–545. https://doi.org/10.1021/acs.estlett.7b00393.

(6) Sumlin, B. J.; Heinson, Y. W.; Shetty, N.; Pandey, A.; Pattison, R. S.; Baker, S.; Hao, W. M.; Chakrabarty, R. K. UV–Vis–IR Spectral Complex Refractive Indices and Optical Properties of Brown Carbon Aerosol from Biomass Burning. *J. Quant. Spectrosc. Radiat. Transf.* **2018**, *206*, 392–398. https://doi.org/10.1016/j.jqsrt.2017.12.009.

(7) Sumlin, B. J.; Oxford, C. R.; Seo, B.; Pattison, R. R.; Williams, B. J.; Chakrabarty, R. K. Density and Homogeneous Internal Composition of Primary Brown Carbon Aerosol. *Environ. Sci. Technol.* **2018**, *52* (7), 3982–3989. https://doi.org/10.1021/acs.est.8b00093.

(8) Carrico, C. M.; Capek, T. J.; Gorkowski, K. J.; Lam, J. T.; Gulick, S.; Karacaoglu, J.; Lee, J. E.; Dungan, C.; Aiken, A. C.; Onasch, T. B.; Freedman, A.; Mazzoleni, C.; Dubey, M. K. Humidified Single-Scattering Albedometer (H-CAPS-PM $_{SSA}$): Design, Data Analysis, and Validation. *Aerosol Sci. Technol.* **2021**, *55* (7), 749–768. https://doi.org/10.1080/02786826.2021.1895430.

(9) Gorkowski, K.; Benedict, K. B.; Carrico, C. M.; Dubey, M. K. Complexities in Modeling Organic Aerosol Light Absorption. *J. Phys. Chem. A* **2022**, *126* (29), 4827–4833. https://doi.org/10.1021/acs.jpca.2c02236.

(10) Saseendran, A.; John, S.; Pakkattil, A.; Thomas, A. P.; Adiga, S.; Chen, J.; Mazzoleni, C.; Viswanath, D.; Varma, R. Retrieval of Broadband Optical Properties from Ambient Aerosols Measurements Using Inverse Mie Calculations. *Aerosol Sci. Eng.* **2022**, *6* (1), 111–125. https://doi.org/10.1007/s41810-021-00128-z.

(11) Washenfelder, R. A.; Flores, J. M.; Brock, C. A.; Brown, S. S.; Rudich, Y. Broadband Measurements of Aerosol Extinction in the Ultraviolet Spectral Region. *Atmospheric Meas. Tech.* **2013**, *6* (4), 861–877. https://doi.org/10.5194/amt-6-861-2013.

(12) Bluvshtein, N.; Lin, P.; Flores, J. M.; Segev, L.; Mazar, Y.; Tas, E.; Snider, G.; Weagle, C.; Brown, S. S.; Laskin, A.; Rudich, Y. Broadband Optical Properties of Biomass-burning Aerosol and Identification of Brown Carbon Chromophores. *J. Geophys. Res. Atmospheres* **2017**, *122* (10), 5441–5456. https://doi.org/10.1002/2016JD026230.

(13) Calderon-Arrieta, D.; Morales, A. C.; Hettiyadura, A. P. S.; Estock, T. M.; Li, C.; Rudich, Y.; Laskin, A. Enhanced Light Absorption and Elevated Viscosity of Atmospheric Brown Carbon through Evaporation of Volatile Components. *Environ. Sci. Technol.* **2024**, *58* (17), 7493–7504. https://doi.org/10.1021/acs.est.3c10184.

(14) Hettiyadura, A. P. S.; Garcia, V.; Li, C.; West, C. P.; Tomlin, J.; He, Q.; Rudich, Y.; Laskin, A. Chemical Composition and Molecular-Specific Optical Properties of Atmospheric Brown Carbon Associated with Biomass Burning. *Environ. Sci. Technol.* **2021**, *55* (4), 2511–2521. https://doi.org/10.1021/acs.est.0c05883.

(15) Lin, P.; Fleming, L. T.; Nizkorodov, S. A.; Laskin, J.; Laskin, A. Comprehensive Molecular Characterization of Atmospheric Brown Carbon by High Resolution Mass Spectrometry with Electrospray and Atmospheric Pressure Photoionization. *Anal. Chem.* **2018**, *90* (21), 12493–12502. https://doi.org/10.1021/acs.analchem.8b02177.

(16) Chen, K.; Raeofy, N.; Lum, M.; Mayorga, R.; Woods, M.; Bahreini, R.; Zhang, H.; Lin, Y.-H. Solvent Effects on Chemical Composition and Optical Properties of Extracted Secondary Brown Carbon Constituents. *Aerosol Sci. Technol.* **2022**, *56* (10), 917–930. https://doi.org/10.1080/02786826.2022.2100734.

(17) Bateman, A. P.; Walser, M. L.; Desyaterik, Y.; Laskin, J.; Laskin, A.; Nizkorodov, S. A. The Effect of Solvent on the Analysis of Secondary Organic Aerosol Using Electrospray Ionization Mass Spectrometry. *Environ. Sci. Technol.* **2008**, *42* (19), 7341–7346. https://doi.org/10.1021/es801226w.

(18) Siemens, K.; Paik, T.; Li, A.; Rivera-Adorno, F.; Tomlin, J.; Xie, Q.; Chakrabarty, R. K.; Laskin, A. Light Absorption and Chemical Composition of Brown Carbon Organic

Aerosol Produced from Burning of Selected Biofuels. *ACS Earth Space Chem.* **2024**, *8* (7), 1416–1428. https://doi.org/10.1021/acsearthspacechem.4c00056.
(19) Chen, Y.; Bond, T. C. Light Absorption by Organic Carbon from Wood Combustion. *Atmospheric Chem. Phys.* **2010**, *10* (4), 1773–1787. https://doi.org/10.5194/acp-10-1773-2010.
(20) Hettiyadura, A. P. S.; Laskin, A. Quantitative Analysis of Polycyclic Aromatic Hydrocarbons Using High-performance Liquid Chromatography-photodiode Array-high-resolution Mass Spectrometric Detection Platform Coupled to Electrospray and Atmospheric Pressure Photoionization Sources. *J. Mass Spectrom.* **2022**, *57* (2), e4804. https://doi.org/10.1002/jms.4804.
(21) Pluskal, T.; Castillo, S.; Villar-Briones, A.; Orešič, M. MZmine 2: Modular Framework for Processing, Visualizing, and Analyzing Mass Spectrometry-Based Molecular Profile Data. *BMC Bioinformatics* **2010**, *11* (1), 395. https://doi.org/10.1186/1471-2105-11-395.
(22) Roach, P. J.; Laskin, J.; Laskin, A. Higher-Order Mass Defect Analysis for Mass Spectra of Complex Organic Mixtures. *Anal. Chem.* **2011**, *83* (12), 4924–4929. https://doi.org/10.1021/ac200654j.
(23) McLafferty, F. W.; Turecek, F. *Interpretation of Mass Spectra: Zahlr. Tab*, 4. Aufl.; University Science Books: Mill Welley, 1993.
(24) Li, Y.; Pöschl, U.; Shiraiwa, M. Molecular Corridors and Parameterizations of Volatility in the Chemical Evolution of Organic Aerosols. *Atmospheric Chem. Phys.* **2016**, *16* (5), 3327–3344. https://doi.org/10.5194/acp-16-3327-2016.
(25) Koch, B. P.; Dittmar, T. From Mass to Structure: An Aromaticity Index for High-resolution Mass Data of Natural Organic Matter. *Rapid Commun. Mass Spectrom.* **2016**, *30* (1), 250–250. https://doi.org/10.1002/rcm.7433.
(26) Calderon-Arrieta, D.; Knull, J.; Xie, Q.; Li, C.; Wang, J.; Evans, L.; Hajian, N.; Hill, K.; Rudich, Y.; Laskin, A. Photolytic Transformation of Soluble and Colloidal Components in Atmospheric Brown Carbon. *ACS EST Air* **2025**, acsestair.5c00301. https://doi.org/10.1021/acsestair.5c00301.
(27) Xie, Q.; Gerrebos, N. G. A.; Calderon-Arrieta, D.; Morton, I. S.; Halpern, E. R.; Li, C.; Zeng, M. F.; Bertram, A. K.; Rudich, Y.; Laskin, A. Molecular Insights into Gas–Particle Partitioning and Viscosity of Atmospheric Brown Carbon. *Environ. Sci. Technol.* **2024**, *58* (41), 18284–18294. https://doi.org/10.1021/acs.est.4c05650.
(28) Xie, Q.; Windwer, E.; Morton, I. S.; Lavin, K. E.; Halpern, E. R.; Nissenbaum, D.; Nizkorodov, S. A.; Rudich, Y.; Laskin, A. Molecular Characterization of Composition and Volatility of Ambient Organic Aerosol Sampled by an UAV-Mounted Portable Aethalometer. *Anal. Chem.* **2025**, *97* (32), 17743–17751. https://doi.org/10.1021/acs.analchem.5c03027.
(29) Li, Y.; Pöschl, U.; Shiraiwa, M. Molecular Corridors and Parameterizations of Volatility in the Chemical Evolution of Organic Aerosols. *Atmospheric Chem. Phys.* **2016**, *16* (5), 3327–3344. https://doi.org/10.5194/acp-16-3327-2016.
(30) West, C. P.; Hsu, Y.-J.; MacFeely, K. T.; Huston, S. M.; Aridjis-Olivos, B. P.; Morales, A. C.; Laskin, A. Volatility Measurements of Individual Components in Organic Aerosol Mixtures Using Temperature-Programmed Desorption–Direct Analysis in Real Time–High Resolution Mass Spectrometry. *Anal. Chem.* **2023**, *95* (19), 7403–7408. https://doi.org/10.1021/acs.analchem.3c00923.
(31) Ranjan, M.; Presto, A. A.; May, A. A.; Robinson, A. L. Temperature Dependence of Gas–Particle Partitioning of Primary Organic Aerosol Emissions from a Small Diesel Engine. *Aerosol Sci. Technol.* **2012**, *46* (1), 13–21. https://doi.org/10.1080/02786826.2011.602761.

(32) Xie, Q.; Halpern, E. R.; Zhang, J.; Shrivastava, M.; Zelenyuk, A.; Zaveri, R. A.; Laskin, A. Volatility Basis Set Distributions and Viscosity of Organic Aerosol Mixtures: Insights from Chemical Characterization Using Temperature-Programmed Desorption–Direct Analysis in Real-Time High-Resolution Mass Spectrometry. *Anal. Chem.* **2024**, *96* (23), 9524–9534. https://doi.org/10.1021/acs.analchem.4c01003.

(33) Xie, Q.; Gerrebos, N. G. A.; Calderon-Arrieta, D.; Morton, I. S.; Halpern, E. R.; Li, C.; Zeng, M. F.; Bertram, A. K.; Rudich, Y.; Laskin, A. Molecular Insights into Gas–Particle Partitioning and Viscosity of Atmospheric Brown Carbon. *Environ. Sci. Technol.* **2024**, *58* (41), 18284–18294. https://doi.org/10.1021/acs.est.4c05650.

(34) Ziemann, P. J.; Atkinson, R. Kinetics, Products, and Mechanisms of Secondary Organic Aerosol Formation. *Chem. Soc. Rev.* **2012**, *41* (19), 6582. https://doi.org/10.1039/c2cs35122f.

(35) Orlando, J. J.; Tyndall, G. S.; Wallington, T. J. The Atmospheric Chemistry of Alkoxy Radicals. *Chem. Rev.* **2003**, *103* (12), 4657–4690. https://doi.org/10.1021/cr020527p.

(36) Pratley, C.; Fenner, S.; Murphy, J. A. Nitrogen-Centered Radicals in Functionalization of Sp^2 Systems: Generation, Reactivity, and Applications in Synthesis. *Chem. Rev.* **2022**, *122* (9), 8181–8260. https://doi.org/10.1021/acs.chemrev.1c00831.

(37) Moise, T.; Flores, J. M.; Rudich, Y. Optical Properties of Secondary Organic Aerosols and Their Changes by Chemical Processes. *Chem. Rev.* **2015**, *115* (10), 4400–4439. https://doi.org/10.1021/cr5005259.
